# Supplementary material for: DORIS study: domestic violence in orthopaedics, a prospective cohort study at a Swedish hospital on the annual prevalence of domestic violence in orthopaedic emergency care
Source: BMJ Open. 2024 May 7;14(5):e085618. doi: 10.1136/bmjopen-2024-085618 (PMC11086206; doi:10.1136/bmjopen-2024-085618)
Supplement: Supplementary data [file bmjopen-2024-085618supp002.pdf]

DORIS

In order to complete the study in the most optimal way, we would be grateful if you would do your best to answer all the questions.

What we mean by violence in close relationships is the emotional, physical or sexual violence that someone you feel emotionally close to (in the household, family or partner relationship) has subjected you to.

Answer using answer sheet A (yellow):

1. **How long have you been together with your current partner?**
(0) Less than 1 year (1) 1-5 years (2) 6-10 years (3) More than 10 years (4) I have no partner

If you have answered ***that you do not have a partner*** *you can skip to question 7*

2. **Is your current partner biologically:**
(0) Man (1) Woman (2) I prefer not to answer

3. **Has your current partner ever subjected you to physical violence?**
Physical violence includes, for example, pushing, hitting, scratching.
(0) Never (1) Occasionally (2) Often

4. **Has your current partner ever subjected you to emotional violence?**
Emotional violence includes, for example, threats, insults, controlling of social contacts.
(0) Never (1) Occasionally (2) Often

5. **Has your current partner ever subjected you to sexual violence?**
Sexual violence includes, for example, sexual humiliation, assault, rape.
(0) Never (1) Occasionally (2) Often

6. **Are you currently seeking medical care for an injury caused by your *current* partner?**
(0) Yes (1) No (2) I prefer not to answer

7. **Are you currently seeking medical care for an injury caused by violence from another person?**
(0) Yes (1) No (2) I prefer not to answer

8. **If you answered yes, what is your relationship with the person who committed the violence?**

9. **Was the person who committed the violence under the influence of any substance when they hurt you?**
(0) Yes, alcohol (1) Yes, drugs (2) I do not know (3) No

10. **Have you previously been seeking medical care for an injury caused by violence in a close relationship?**
(0) Yes: (*circle this if it happened in the current/past relationship)* (1) No (2) I prefer not to answer

11. **Do you think it is important that health care services ask about violence in close relationships?**
(0) Yes (1) No

12. **What is your level of education?**
(0) Primary school (1) High School (2) College/University

13. If you have been subjected to violence in a **current** close relationship, you have the option to receive counselling with a social worker at the trauma center at Mölndal Hospital. You will be called for a return visit in 1-2 weeks and the social worker's visit will not be visible in your medical record or on the call on paper you receive at home. The call will look like a regular call for a medical appointment. If your injury also requires a medical follow-up, you will be scheduled to see the social worker after your medical appointment. The social worker is subject to existing healthcare laws. Do you wish to speak to a social worker?

DORIS

Answer sheet A
please check the option that best applies to you

---

1. [0] Less than 1 year [1] 1-5 years [2] 6-10 years [3] More than 10 years [4] I have no partner

If you have answered ***that you do not have a partner*** *you can skip to question 7!*

2. [0] Man [1] Woman [2] I prefer not to answer
3. [0] Never [1] Occasionally [2] Often
4. [0] Never [1] Occasionally [2] Often
5. [0] Never [1] Occasionally [2] Often
6. [0] Yes [1] No [2] I prefer not to answer
7. [0] Yes [1] No [2] I prefer not to answer
8. ____
9. [0] Yes, alcohol [1] Yes, drugs [2] I do not know [3] No
10. [0] Yes: currently / in the past [1] No [2] I prefer not to answer

    If you have answered yes, how many times have you been seeking medical care: ___________

11. [0] Yes [1] No
12. [0] Primary school [1] High School [2] College/University

13. [0] Yes [1] No

Consent to the DORIS study
I have been provided with the written information and hereby consent to the processing of my information in the DORIS study as described in the information for research participants.

__________________________________ __________________________________
Signature Clarification of signature

__________________________________ ____________________
National identification number Date

DORIS

Questionnaire B

What we mean by violence in close relationships is the emotional, physical or sexual violence that someone you feel emotionally close to (in the household, family or partner relationship) has subjected you to.

1. Are you currently seeking medical care for an injury caused by violence in a close relationship?

YES NO

2. Do you think that health care services should routinely ask about violence in close relationships as the cause of injuries?

YES NO

DORIS

Answer sheet B
please circle the answer that best applies to you

1. YES NO

2. YES NO

DORIS

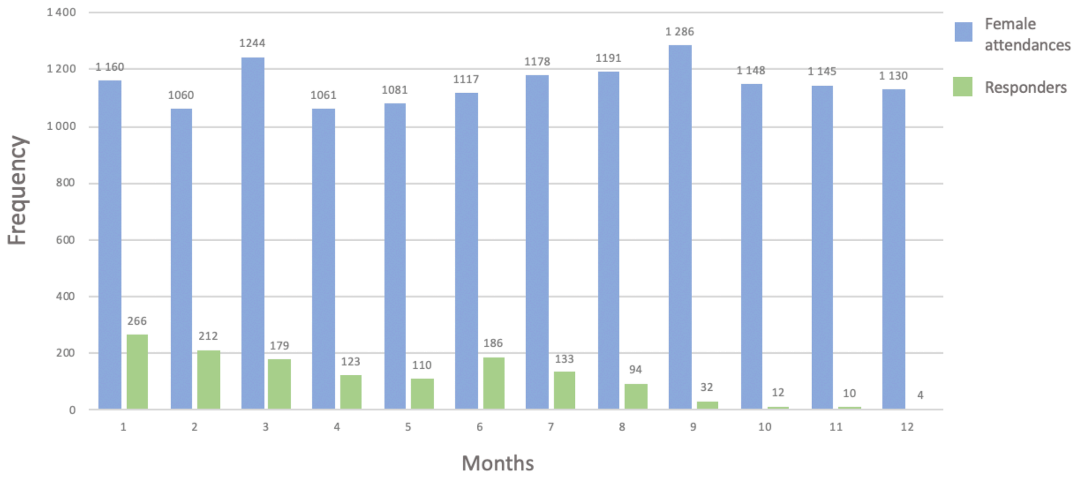

Supplementary material, Figure S1. The responder frequency per month The number of unique female attendances (blue) and responders (green) per study month (21st to 21st of the next calendar month).
