# Supplementary material for: DORIS study: domestic violence in orthopaedics, a prospective cohort study at a Swedish hospital on the annual prevalence of domestic violence in orthopaedic emergency care
Source: BMJ Open. 2024 May 7;14(5):e085618. doi: 10.1136/bmjopen-2024-085618 (PMC11086206; doi:10.1136/bmjopen-2024-085618)
Supplement: Author's manuscript [file bmjopen-2024-085618.draft_revisions.pdf]

BMJ Open is committed to open peer review. As part of this commitment we make the peer review history of every article we publish publicly available.

When an article is published we post the peer reviewers' comments and the authors' responses online. We also post the versions of the paper that were used during peer review. These are the versions that the peer review comments apply to.

The versions of the paper that follow are the versions that were submitted during the peer review process. They are not the versions of record or the final published versions. They should not be cited or distributed as the published version of this manuscript.

BMJ Open is an open access journal and the full, final, typeset and author-corrected version of record of the manuscript is available on our site with no access controls, subscription charges or pay-per-view fees (http://bmjopen.bmj.com).

If you have any questions on BMJ Open's open peer review process please email info.bmjopen@bmj.com

The DORIS study: Domestic violence in ORthopaedIcS, an observational study of 1,366 female patients

Journal:	*BMJ Open*
Manuscript ID	bmjopen-2024-085618
Article Type:	Original research
Date Submitted by the Author:	21-Feb-2024
Complete List of Authors:	Svensson Malchau, Karin; Sahlgrenska University Hospital, Orthopaedics<br>Caragounis, Eva-Corina; University of Gothenburg, Department of Surgery, Institute of Clinical Sciences<br>Sundfeldt, Mikael; Sahlgrenska University Hospital, Orthopaedics
Keywords:	ORTHOPAEDIC & TRAUMA SURGERY, ACCIDENT & EMERGENCY MEDICINE, Awareness, Health, Mass Screening

SCHOLARONE™
Manuscripts

I, the Submitting Author has the right to grant and does grant on behalf of all authors of the Work (as defined in the below author licence), an exclusive licence and/or a non-exclusive licence for contributions from authors who are: i) UK Crown employees; ii) where BMJ has agreed a CC-BY licence shall apply, and/or iii) in accordance with the terms applicable for US Federal Government officers or employees acting as part of their official duties; on a worldwide, perpetual, irrevocable, royalty-free basis to BMJ Publishing Group Ltd ("BMJ") its licensees and where the relevant Journal is co-owned by BMJ to the co-owners of the Journal, to publish the Work in this journal and any other BMJ products and to exploit all rights, as set out in our licence.

The Submitting Author accepts and understands that any supply made under these terms is made by BMJ to the Submitting Author unless you are acting as an employee on behalf of your employer or a postgraduate student of an affiliated institution which is paying any applicable article publishing charge ("APC") for Open Access articles. Where the Submitting Author wishes to make the Work available on an Open Access basis (and intends to pay the relevant APC), the terms of reuse of such Open Access shall be governed by a Creative Commons licence – details of these licences and which Creative Commons licence will apply to this Work are set out in our licence referred to above.

Other than as permitted in any relevant BMJ Author's Self Archiving Policies, I confirm this Work has not been accepted for publication elsewhere, is not being considered for publication elsewhere and does not duplicate material already published. I confirm all authors consent to publication of this Work and authorise the granting of this licence.

For peer review only

The DORIS study: Domestic violence in ORthopaedIcS, an observational study of 1,366 female patients

Karin Svensson Malchau MD PhD[1], Eva-Corina Caragounis MD PhD[2], Mikael Sundfeldt MD PhD[1]

[1]Department of Orthopaedics, Institute of Clinical Sciences, Sahlgrenska University Hospital, Sahlgrenska Academy, University of Gothenburg, Gothenburg, Sweden
[2]Department of Surgery, Institute of Clinical Sciences, Sahlgrenska University Hospital, Sahlgrenska Academy, University of Gothenburg, Gothenburg, Sweden

Corresponding author: Karin Svensson Malchau, Department of Orthopaedics, R-huset, Länsmansvägen 28, 431 42 Mölndal, Sweden. E-mail address: karin.am.svensson@vgregion.se, telephone number +46(0)313430761.

Word count: 2,568

For peer review only

For peer review only - http://bmjopen.bmj.com/site/about/guidelines.xhtml

ABSTRACT

Background

Domestic violence (DV) is a major problem which despite many efforts persists globally. Victims of DV can present with various injuries, whereof musculoskeletal presentation is common. Injury severity may escalate as DV continues, thus making early identification and intervention, within a healthcare setting, an important strategy in the work against DV.

Objectives

The DORIS study aimed to establish the year prevalence of DV at an orthopaedic emergency department (ED) in Sweden. The prevalence of injuries due to DV and current experience of any type of DV was surveyed.

Design

Female adult patients with orthopaedic injuries seeking treatment at a tertiary orthopaedic centre received forms containing validated questions for DV between September 2021–2022 during their ED visit. Furthermore, all cases of DV identified by staff at the ED were reported. Affected patients were offered to see a counsellor within the project.

Results

During the study period, 4,192 female patient were provided with study forms and 1,366 responded (32.5%). One in 14 had experience of current DV (n=100, 7.5%) and one in 65 (n=21, 1.5%) had an injury due to DV. Of patients injured due to DV, 50 % had previously sought medical attention. Formal documentation of DV was missing in 50 % of the cases, and these cases had been identified thanks to screening.

Conclusions

The prevalence of DV found in the current study is comparable to international findings and adds to the growing body of evidence that it needs to be considered in clinical practice. It is

DORIS

important to raise awareness of DV, and frame strategies, as health caregivers have a unique position to identify and offer intervention to DV victims.

ARTICLE SUMMARY

Strengths and limitations of this study

- This is a prospective observational study investigating the year prevalence of domestic violence (DV) in female orthopaedic patients using questionnaires containing validated questions for DV.
- Study participants were approached individually without the presence of company and great discretion was taken to ascertain the safety of DV victims.
- The study was designed to screen all female patients consecutively, and although difficulties in the practical implementation of the screening program impeded the desired inclusion rate, a large volume of patients were included.
- Study participants could not chose to be anonymous which may have deterred some patients from filling out the study questionnaires.

Keywords: orthopaedics, domestic violence, trauma

only

For peer review only - http://bmjopen.bmj.com/site/about/guidelines.xhtml

DORIS

Introduction

Domestic violence (DV) is a serious public health problem estimated to affect as many as 27% of women in partner relationships during their lifetime.[1] It is an insidious process, starting off with phases of systematic psychological abuse often leading to physical abuse.[2] Aside its societal and individual economic consequences,[3] it is one of the most common causes for physical injuries in women and victims are at great risk for mental health issues, suicide and homicide.[4] 20–50% of female homicides, are caused by a former or current intimate partner,[5, 6] and in Sweden, the death toll due to known DV was 13 in 2020.[7]

Musculoskeletal injuries are one of the most common presentations of DV.[8, 9] One in 50 women present to fracture clinics with an injury due to DV.[10] Recognition of DV as an injury mechanism is important and orthopaedic units have been suggested ideal for screening.[11, 12] However, the difficulties of identifying DV are many. Victims may be prevented from seeking medical attention which was found true for 36% of women in Canada.[8] A further challenge is the absence of active questioning in healthcare and that patients may not disclose occurrence of abuse.[13] Orthopaedic surgeons under-estimate the prevalence of DV,[14] and do not ask about DV.[10]

Implementation of screening within healthcare may lead to a greater detection of DV, which in turn can be potentially lifesaving. Nevertheless, questioning for DV is not standard and formal documentation is poor.[15] Sweden is considered the most gender equal country in the European Union,[7] however, research on DV in orthopaedics is scarce and little is known about its prevalence in Sweden. The current project aimed to identify the year prevalence of orthopaedic injuries caused by DV, and current experience of DV, in female patients at the largest orthopaedic emergency department (ED) in Sweden.

For peer review only - http://bmjopen.bmj.com/site/about/guidelines.xhtml

DORIS

Methods

Study design

This is a self-reported questionnaire-based study including questions validated for detection of partner violence in an orthopaedic setting.[11]

Setting

The study was conducted at the ED of the Sahlgrenska University Hospital/Mölndal in Gothenburg, Sweden from 21st September 2021 to 21st September 2022. The ED averages 45,000 unique attendances yearly and the orthopaedic section has an average of 38 female attendances daily.

Sets of study information, marked with name and social security number, were assembled upon triage. Staff were instructed to hand out the forms to all female patients fulfilling the study inclusion criteria. Forms were handed out in the examination room to fill out in private and put in a sealed envelope (Figure 1). ED staff were unaware of status of study participation. The forms were contained inside the ED as a precautious measure to diminish the risk of unauthorized persons identifying potential victims. There were two forms (A and B) of which B was simplified to encourage higher responder rates (Supplementary material, S1). If ED staff discovered a case of DV when informing patients about the study, they were asked to mark the envelope with an "X". However, the patient was only included in the further analysis if she consented to study participation. Medical records of consenting patients reporting DV were reviewed to assess injury type and severity.

For peer review only - http://bmjopen.bmj.com/site/about/guidelines.xhtml

Patients who wished to meet a project counsellor were booked for a medical follow-up without mention of the counsellor. This was intentional to protect the patient in cases of cohabitation.

Participants

Patients of female sex of at least 18 years of age and with residency in Sweden triaged to the orthopaedic section of the ED were included in the study. Study forms were provided in Swedish and translated two-way in English and Arabic. Patients accompanied by someone, or with cognitive impairment or physical impairment, i.e. dementia or poor eyesight, were excluded. No sample size calculation was conducted as the objective was to establish the year prevalence of DV victims.

Objectives

The primary objective was to identify the prevalence of orthopaedic injuries sustained directly due to DV. The secondary objective was to establish the prevalence of current experience of DV.

Definitions

DV was defined as emotional, physical or sexual abuse. Any occurrence within the family, domestic unit or by former intimate partners, was included, as defined by the Istanbul Convention (2011).[16] A relationship was defined as a partnership lasting at least one month.

Data analysis

Data was analysed descriptively with frequency counts and percentages for categorical variables. Software IBM SPSS version 29 was used for data analysis.

For peer review only - http://bmjopen.bmj.com/site/about/guidelines.xhtml

DORIS

Ethical considerations

Written consent was obtained upon study enrolment in line with the Ethical Review Board's regulations (DNR 2021-01752).

Patient and Public Involvement

It was not deemed appropriate to involve patients or the public in the design, or conduct, or reporting, or dissemination plans of our research.

Results

In total, 4,192 (30.4%) out of 13,801 unique female attendances registered at the orthopaedic section of the ED were given study forms. Of these, 1,366 (32.6%) agreed to inclusion (Figure 2). The majority of responders spoke Swedish (99.4%), did not live in a socially disadvantaged area (80.4%) and were in a relationship (62.2%) (Table 1).

Experience of DV

Of the 1,366 patients, 100 patients (7.5%) had current experience of DV and 21 (1.5%) of them had an injury due to DV. Of the 21 patients, 16 consented to filling out the study forms. The remaining 5 patients disclosed DV to healthcare staff, but declined to fill out the study forms and were not included in the further analysis, leaving 95 patients for further analysis (Figure 2).

For peer review only

DORIS

DV (any type) was reported by 89 (89/1361, 6.5%) patients in their current relationship. Emotional abuse was most common (69/89, 77.5%) followed by physical abuse (33/89, 37.1%) and sexual abuse (19/89, 21.3%) (Figure 3).

DV as a direct cause of injury

In total, 21 patients with an injury due to DV were identified (Figure 2), meaning that one in 65 patients needed medical attention due to physical abuse. Of the 16 consenting DV victims, 8 had previously been in contact with healthcare for an injury due to abuse. Formal documentation of DV was noted in 8 medical records, and in the remaining cases the injury mechanism was unspecified fall trauma (Table 2).

The age span of DV victims was 18–76 years. 3 patients were from socially disadvantaged areas and 3 patients had female partners. The majority of patients had completed high school, but had no further academic education (Table 1). 8 patients reported on repeated abuse in their current relationship of which 5 stated an occurrence of both emotional, physical and sexual abuse.

Fractures were the most prevalent injury followed by contusions and joint distortions (Table 3). Five patients sustained injuries requiring sick leave and 2 patients required surgery (Table 3). Thirty-seven follow-up visits were recorded due to DV injuries (excluding visits to the counsellor).

Screening for DV

In total, 1208 women (89.0%) were of the opinion that healthcare staff should ask about DV (Table 1). However, 2 of the 16 patients (12.5%) injured due to DV did not feel that screening

For peer review only

For peer review only - http://bmjopen.bmj.com/site/about/guidelines.xhtml

DORIS

was necessary. Fifty-four patients (4.0%) had previously contacted healthcare for physical abuse (Table 1), whereof 34 of these patients were still in an abusive relationship.

The project counsellors had contact with 23 patients whereof 8 had been injured due to DV. 12 patients (52.2%) showed up for their appointment. 6 patients failed to appear, 4 had misunderstood or were too injured to come for their appointment. 1 patient had given a faulty address and did not respond to phone calls.

Discussion

The DORIS study aimed to establish the prevalence of current experience of DV, and injuries directly caused by DV, in female patients in the largest orthopedic ED in Northern Europe. A rate of one in 14 patients (100/1366, 7.5%) with current experience of DV and one in 65 patients (21/1366, 1.6%) injured due to DV was established.

The prevalence of injuries due to DV is comparable with the results of a multi-national investigation in orthopedic injury clinics, conducted by the PRAISE group, of 0-3%.[10] Previous research has reported a 12-month prevalence of DV of 15–22% in orthopedic patients.[10, 17] The 12-month prevalence was not investigated in the DORIS study. However, 6.5% (89/1366) experienced DV in a current partner relationship. This excludes current experience of DV carried out by a family member or former partner which may explain the relatively low prevalence. Differences in recruitment methods, study settings and staff engagement could be a further explanation. The lower prevalence may also reflect governmental and societal policies on gender equality in Sweden.

For peer review only - http://bmjopen.bmj.com/site/about/guidelines.xhtml

DORIS

When comparing proportions of type of abuse, the present study established that emotional abuse was most common. This is also true in Scottish, American and Canadian settings.[10, 17] However, surprisingly, in the Netherlands and in Denmark, countries seemingly more comparable to Sweden, physical abuse was most common.[10] It may be difficult to understand what is meant by emotional abuse, the DORIS study forms contained examples of emotional abuse which may explain the higher prevalence.

Formal documentation of DV was noted in 50% of cases, meaning that 50% were not identified in the regular healthcare setting. Routine screening of DV leads to higher detection rates,[18] however, only 2% of healthcare workers in orthopedics routinely ask about it.[19] Surgeons feel uncomfortable and unsure of what to do if their patient is a victim which calls for better education and support models within healthcare.[20, 21]

Although it is important to be suspicious of inconsistent injury mechanisms or “red flags”, such as falling down the stairs,[22] feasible injury mechanisms were disclosed in 50% of the DV cases. Hence, questioning for DV should not just be conducted when suspicion is raised, as is often the case. Within the DORIS study, direct questioning, in questionnaire format, was used as this has proven efficient for DV screening and is less time consuming in an ED setting.[11, 23] However, the study forms contained a lot of text due to regulations stated by the Ethical Review Board, which may have discouraged potential responders. In the continued work of improving DV detection at the study site efforts will be made to optimise the screening tool.

Merely 50% of patients with an injury due to DV had previously been in contact with healthcare for DV. Hence, the remaining patients may have presented with an index injury. This finding supports the, previously suggested,[11, 19] need for screening in orthopaedic

For peer review only - http://bmjopen.bmj.com/site/about/guidelines.xhtml

settings, as early intervention can be potentially lifesaving. Up to 81% of female patients are of the opinion that healthcare staff should ask about DV.[10, 19, 23] The corresponding numbers were somewhat higher in the DORIS study (89% in the entire cohort and 94-96% in abused patients). Cultural differences and thereby expectations on healthcare may explain the aforementioned span.

The strength of the DORIS study is its setting at the largest orthopaedic ED in Northern Europe. After, the PRAISE study,[10] DORIS is the largest prevalence study in orthopedics. Due to COVID restrictions during the study period, company was generally not allowed in the ED which facilitated the distribution of study forms. Victims of DV were also offered follow-up with a counsellor within the study.

A major limitation may be nonresponse bias. Although the study was regarded important by ED staff, the distribution rate of study forms was 30% and response rate 33%. The authors had meetings with ED staff and two counsellors were recruited to provide an in-house support program to increase the likelihood for staff engagement.[19] Unfortunately, due to management issues, the staffing situation became more turbulent with several experienced nurses and assistant nurses choosing to resign throughout the year. The authors believe that the inconsistencies in staffing were the main reason for poor study enrolment (Supplementary material, Figure 1). In addition, despite being an excellent forum for DV screening,[18] in regard to the “open window phase” (in which victims may be more receptive and prone to seek help after abuse),[24] the ED as such is a busy and stressful place. In general, detecting DV may be difficult in such a setting: staff may be unaware of DV as a problem, and patients may feel uncomfortable confiding in ED staff. For this reason, it is crucial to structure EDs in a

For peer review only

manner where triage can be done in private, as also suggested by Ahmad et al.,[18] and where patients are unaccompanied in triage as standard routine.

Poor response rate was partly expected. Similar studies,[10, 17] have had different approaches to recruitment making it difficult to evaluate what an acceptable response rate is. Due to the delicate nature of the study, the authors had preferred that social security number and further personal details were omitted when consenting to the study. The need to do this may have deterred potential victims from disclosing DV. However, full disclosure of personal details was a requirement from the Ethical Review Board due to research regulations. Furthermore, the authors have reason to believe that the 2,325 patients who for some reason did not wish to participate in the study may not have received proper study information or been given a chance to fill out the study forms.

The exclusion criteria imply certain limitations. Elderly patients, either accompanied by caregivers or with the diagnosis of dementia, were not included. Despite the difficulties of capturing cases in this group, it is important to acknowledge their vulnerability and that both dementia and female sex are predictive of abuse.[25] Furthermore, the authors acknowledge that DV affects both female and male patients. Screening of females was chosen as female DV patients have a greater fracture risk, 83% of ED visits due to DV are female, and 50% of female homicides are due to DV.[12] However, the long-term goal for the DORIS project is to provide a healthcare program dedicated to DV patients regardless of sex.

The DORIS study focused on current abuse, whereas previous research, such as conducted by the PRAISE group and Sardinha et al. also investigated life-time abuse.[1, 10, 17] In hindsight, the inclusion of life-time abuse would have been interesting for comparative reasons. However,

For peer review only - http://bmjopen.bmj.com/site/about/guidelines.xhtml

when designing the study, the authors decided that the patient's current situation was the most clinically relevant and therefore most important.

Despite its limitations, and a probable under-reporting of DV, the finding of one in 65 patients translates to one victim of DV injuries nearly every second day, and two to three patients with current experience of DV daily, at the study center. Interventions are essential to disrupt continued abuse and healthcare has an important role in the detection of DV.[9, 26] The experience generated by the present study suggests that screening is necessary in order to improve identification of DV cases and that patients expect healthcare to engage in detecting DV. The results from the DORIS study will be used to improve routines at the study site, and hopefully inspire to similar actions elsewhere.

Conclusion

The prevalence of DV established in the current study implies a high annual volume of DV victims at the study site. DV victims may come to an orthopaedic setting with an index injury and healthcare staff have an unique opportunity to intervene. The DORIS study adds to the growing body of evidence that DV needs attention in the healthcare setting. Increased awareness and actions to identify DV is imperative and it is important to educate, engage and provide adequate conditions for healthcare staff to conduct screening. Future work should focus on implementing DV screening as a routine and provide a safe environment for DV victims in all healthcare disciplines.

Author contributions

For peer review only - http://bmjopen.bmj.com/site/about/guidelines.xhtml

KSM participated in the planning and design of the study, collection of patient data, analyzed the data, interpreted the data, drafted the manuscript and critically revised the manuscript. ECC participated in the planning and design of the study and critically revised the manuscript. MS participated in the planning and design of the study and critically revised the manuscript.

Acknowledgements

The authors would like to thank Sandra Rosnell, Linda Stolpe, Stella Sundfeldt, Ann-Christin von Corswant, Anne Louise Gidestrand, the medical staff at the ED at Mölndal's Hospital and all the patients who chose to participate.

Funding

The study was funded by Doktor Felix Neuberghs stiftelse, BGS forskningsstipendium, Göteborgs Läkaresällskap, SU-fonderna, Konrad och Helfrid Johanssons stiftelse.

Competing interest statement

All authors have completed the ICMJE uniform disclosure form at http://www.icmje.org/disclosure-of-interest/ and declare: no support from any organisation for the submitted work; no financial relationships with any organisations that might have an interest in the submitted work in the previous three years; no other relationships or activities that could appear to have influenced the submitted work

References

1. Sardinha L, Maheu-Giroux M, Stockl H, Meyer SR, Garcia-Moreno C. Global, regional, and national prevalence estimates of physical or sexual, or both, intimate partner violence against women in 2018. Lancet. 2022;399(10327):803-13.
2. Murphy CM, O'Leary KD. Psychological aggression predicts physical aggression in early marriage. J Consult Clin Psychol. 1989;57(5):579-82.

For peer review only

3. Max W, Rice DP, Finkelstein E, Bardwell RA, Leadbetter S. The economic toll of intimate partner violence against women in the United States. Violence Vict. 2004;19(3):259-72.

4. Ellsberg M, Jansen HA, Heise L, Watts CH, Garcia-Moreno C, Health WHOM-cSoWs, et al. Intimate partner violence and women's physical and mental health in the WHO multi-country study on women's health and domestic violence: an observational study. Lancet. 2008;371(9619):1165-72.

5. Jack SPD, Petrosky E, Lyons BH, Blair JM, Ertl AM, Sheats KJ, et al. Surveillance for Violent Deaths - National Violent Death Reporting System, 27 States, 2015. MMWR Surveill Summ. 2018;67(11):1-32.

6. Stockl H, Devries K, Rotstein A, Abrahams N, Campbell J, Watts C, et al. The global prevalence of intimate partner homicide: a systematic review. Lancet. 2013;382(9895):859-65.

7. [Available from: https://eige.europa.eu/gender-equality-index/2022/domain/violence/SE.

8. Bhandari M, Dosanjh S, Tornetta P, 3rd, Matthews D, Violence Against Women Health Research C. Musculoskeletal manifestations of physical abuse after intimate partner violence. J Trauma. 2006;61(6):1473-9.

9. Giordano V, Giordano C, Lopes IM, Pires RE, Godoy-Santos A, Giannoudis PV. Orthopaedic surgeons can play important role in identifying victims of domestic violence in the emergency department - narrative review of Brazilian literature. Medicine (Baltimore). 2022;101(50):e31461.

10. Investigators P, Sprague S, Bhandari M, Della Rocca GJ, Goslings JC, Poolman RW, et al. Prevalence of abuse and intimate partner violence surgical evaluation (PRAISE) in orthopaedic fracture clinics: a multinational prevalence study. Lancet. 2013;382(9895):866-76.

11. Sprague S, Madden K, Dosanjh S, Petrisor B, Schemitsch EH, Bhandari M. Screening for intimate partner violence in orthopedic patients: a comparison of three screening tools. J Interpers Violence. 2012;27(5):881-98.

12. Loder RT, Momper L. Demographics and Fracture Patterns of Patients Presenting to US Emergency Departments for Intimate Partner Violence. J Am Acad Orthop Surg Glob Res Rev. 2020;4(2).

13. Brown JB, Lent B, Schmidt G, Sas G. Application of the Woman Abuse Screening Tool (WAST) and WAST-short in the family practice setting. J Fam Pract. 2000;49(10):896-903.

14. Bhandari M, Sprague S, Tornetta P, 3rd, D'Aurora V, Schemitsch E, Shearer H, et al. (Mis)perceptions about intimate partner violence in women presenting for orthopaedic care: a survey of Canadian orthopaedic surgeons. J Bone Joint Surg Am. 2008;90(7):1590-7.

15. Khurana B, Sing D, Gujrathi R, Keraliya A, Bay CP, Chen I, et al. Recognizing Isolated Ulnar Fractures as Potential Markers for Intimate Partner Violence. J Am Coll Radiol. 2021;18(8):1108-17.

16. Council of Europe Convention on preventing and combating violence against women and domestic violence. Istanbul; 2011.

17. Downie S, Kanya I, Madden K, Bhandari M, Jariwala AC. Intimate partner violence (IPV) in male and female orthopaedic trauma patients: a multicentre, cross-sectional prevalence study. BMJ Open. 2021;11(8):e046164.

18. Ahmad I, Ali PA, Rehman S, Talpur A, Dhingra K. Intimate partner violence screening in emergency department: a rapid review of the literature. J Clin Nurs. 2017;26(21-22):3271-85.

For peer review only

19. Downie S, Madden K, Bhandari M, Jariwala A. A prospective questionnaire-based study on staff awareness of intimate partner violence (IPV) in orthopaedic trauma patients. Surgeon. 2019;17(4):207-14.
20. Della Rocca GJ, Sprague S, Dosanjh S, Schemitsch EH, Bhandari M. Orthopaedic surgeons' knowledge and misconceptions in the identification of intimate partner violence against women. Clin Orthop Relat Res. 2013;471(4):1074-80.
21. Sprague S, Swinton M, Madden K, Swaleh R, Goslings JC, Petrisor B, et al. Barriers to and facilitators for screening women for intimate partner violence in surgical fracture clinics: a qualitative descriptive approach. BMC Musculoskelet Disord. 2013;14:122.
22. Spedding RL, McWilliams M, McNicholl BP, Dearden CH. Markers for domestic violence in women. J Accid Emerg Med. 1999;16(6):400-2.
23. Furbee PM, Sikora R, Williams JM, Derk SJ. Comparison of domestic violence screening methods: a pilot study. Ann Emerg Med. 1998;31(4):495-501.
24. Curnow SA. The Open Window Phase: helpseeking and reality behaviors by battered women. Appl Nurs Res. 1997;10(3):128-35.
25. Gardezi M, Moore HG, Rubin LE, Grauer JN. Predictors of Physical Abuse in Elder Patients With Fracture. J Am Acad Orthop Surg Glob Res Rev. 2022;6(7).
26. Sprague S, Madden K, Dosanjh S, Godin K, Goslings JC, Schemitsch EH, et al. Intimate partner violence and musculoskeletal injury: bridging the knowledge gap in orthopaedic fracture clinics. BMC Musculoskelet Disord. 2013;14:23.

For peer review only

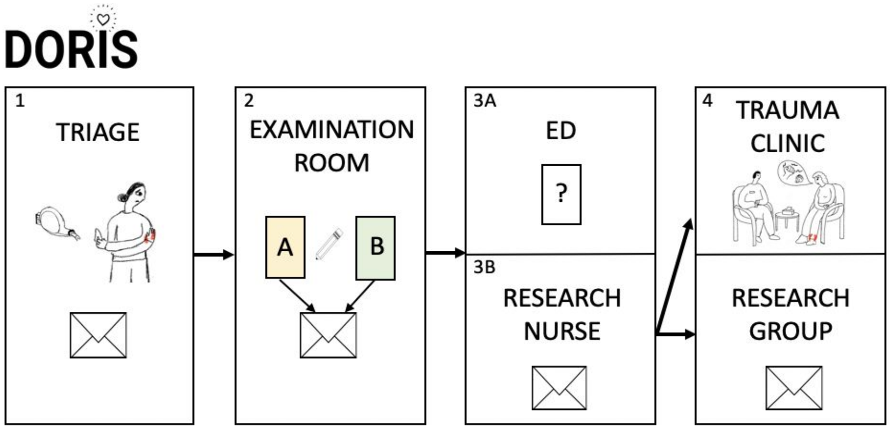

For peer review only

DORIS

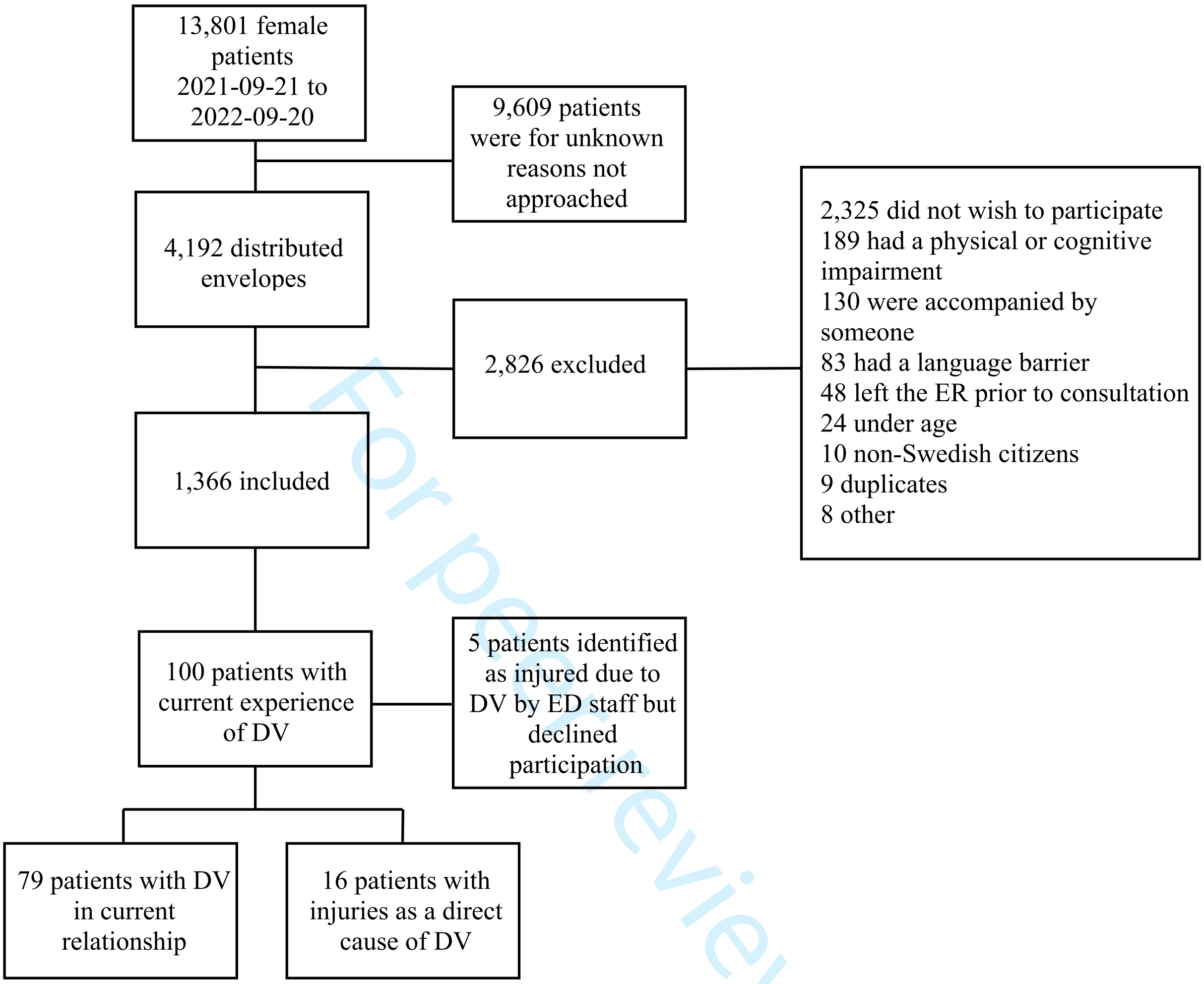

For peer review only

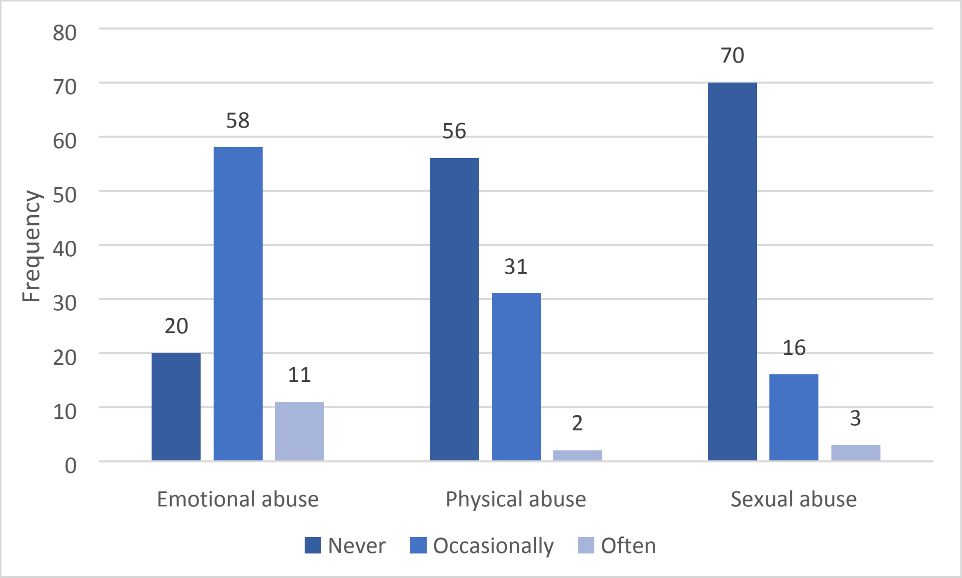

For peer review only

Table 1. Demographics of all responders and whether health care should ask about DV grouped by experience of DV.

	All responders (n=1,361)	Responders reporting no experience of DV in current relationship (n=1,165)	Responders reporting current DV, not DV as direct cause of injury (n=79)	Responders reporting DV as direct cause of injury (n=16)	Responders with missing or incomplete answers on current DV (n=101)
**Age** (range, years)					
18-29	226 (16.6)	201 (17.3)	8 (10.1)	2 (12.5)	15 (14.8)
30-39	211 (15.5)	182 (15.6)	13 (16.5)	4 (25.0)	12 (11.9)
40-49	211 (15.5)	174 (14.9)	17 (21.5)	4 (25.0)	16 (15.8)
50-59	262 (19.3)	234 (20.1)	15 (19.0)	1 (6.3)	12 (11.9)
60-69	239 (17.6)	199 (17.1)	17 (21.5)	4 (25.0)	19 (18.8)
>70	202 (14.8)	167 (14.3)	8 (10.1)	1 (6.3)	26 (25.7)
Missing	10 (0.7)	8 (0.7)	1 (1.3)	0 (0.0)	1 (1.0)
**Resident of a socially disadvantaged area**					
Yes	227 (16.7)	184 (15.8)	16 (20.3)	3 (18.8)	24 (23.8)
No	1101 (80.9)	954 (81.9)	58 (73.4)	13 (81.3)	75 (75.2)
Protected person/not a resident in Gothenburg	9 (0.7)	6 (0.1)	3 (3.8)	0 (0.0)	0 (0.0)
Missing	24 (1.8)	21 (1.8)	2 (2.5)	0 (0.0)	1 (1.0)
**Language**					
Swedish	1353 (99.4)	1161 (99.7)	77 (97.5)	15 (94.1)	100 (99.0)
English	5 (0.4)	3 (0.3)	1 (1.3)	0 (0.0)	1 (1.0)
Arabic	3 (0.2)	1 (0.0)	1 (1.3)	1 (5.9)	0 (0.0)
**Education level**					
Compulsory school	104 (7.6)	82 (7.0)	5 (6.3)	2 (12.5)	15 (14.9)
High school	459 (33.7)	395 (33.9)	30 (38.0)	9 (56.3)	25 (24.8)
University	727 (53.4)	650 (55.8)	43 (54.4)	4 (25.0)	29 (28.7)
Missing	71 (5.3)	38 (3.3)	1 (1.3)	1 (6.3)	31 (30.7)
**Partner sex**					
No partner	430 (31.6)	427 (36.7)	0 (0.0)	3 (17.6)	0 (0.0)
Male	806 (59.2)	711 (61.0)	75 (95.0)	10 (64.7)	10 (10.0)
Female	40 (2.9)	22 (2.0)	2 (2.5)	2 (11.8)	14 (13.9)
Missing	85 (6.2)	5 (0.4)	2 (2.5)	1 (5.9)	77 (76.2)
**Duration of relationship**					
Less than one year	36 (2.6)	30 (2.6)	4 (5.1)	2 (12.5)	1 (1.0)
1-5years	165 (12.1)	144 (12.4)	12 (15.2)	3 (18.8)	6 (5.9)
6-10 years	96 (7.1)	80 (6.9)	12 (15.2)	2 (12.5)	2 (2.0)
More than 10 years	567 (41.7)	476 (40.9)	50 (63.3)	5 (31.3)	36 (35.6)
No partner	440 (32.3)	430 (36.9)	1 (1.3)	3 (18.8)	6 (5.9
Missing	57 (4.2)	5 (0.4)	0 (0.0)	2 (12.5)	50 (50.0)
**Have you ever sought medical care for DV?**					
No	1050 (77.1)	942 (80.9)	57 (72.2)	8 (50.0)	43 (42.6)
Yes	54 (4.0)	38 (3.3)	10 (12.7)	3 (18.8)	4 (4.0)
Missing	257 (18.9)	185 (15.9)	13 (16.5)	5 (31.3)	54 (53.5)
**Should health care workers ask about DV?**					
Yes	1209 (88.8)	1068 (91.7)	76 (96.2)	14 (87.5)	51 (50.5)
No	41 (3.0)	30 (2.6)	2 (2.5)	0 (0.0)	9 (8.9)
Missing	111 (8.2)	67 (5.8)	1 (1.3)	2 (12.5)	41 (40.6)

For peer review only

Table 2. Injury mechanism as stated in the medical records and treatment needs due to DV

	Frequency n (%)
**Formal documentation of DV in medical record**	
Yes	8 (50)
No	8 (50
**Stated injury mechanism in medical record**	
Fall trauma, unspecified	8 (50)
Abuse	8 (50)
**Orthopaedic treatment**	
Pain medication and physiotherapy	8 (50)
Immobilization (cast/orthosis)	6 (38)
Surgery	2 (12)
**Need for sick leave**	
Yes	5 (31)
No	11 (69)

For peer review only

Table 3. Type of injuries noted in cases with DV as direct cause of injury

Injury type and localisation	Frequency n (%)
**Fracture**	6 (38)
Hand	5
Foot	1
**Contusion**	4 (25)
Upper extremity	1
Lower extremity	3
**Distortion**	4 (25)
Shoulder	1
Knee	1
Foot	2
**Joint dislocation**	1 (6)
**Ligament rupture**	1 (6)
**Laterality of injury**	
Right	10 (63)
Left	4 (25)
Missing	2 (12)

For peer review only

DORIS

In order to complete the study in the most optimal way, we would be grateful if you would do your best to answer all the questions.

What we mean by violence in close relationships is the emotional, physical or sexual violence that someone you feel emotionally close to (in the household, family or partner relationship) has subjected you to.

Answer using answer sheet A (yellow):

1. **How long have you been together with your current partner?**
(0) Less than 1 year (1) 1-5 years (2) 6-10 years (3) More than 10 years (4) I have no partner

If you have answered ***that you do not have a partner*** *you can skip to question 7*

2. **Is your current partner biologically:**
(0) Man (1) Woman (2) I prefer not to answer

3. **Has your current partner ever subjected you to physical violence?**
Physical violence includes, for example, pushing, hitting, scratching.
(0) Never (1) Occasionally (2) Often

4. **Has your current partner ever subjected you to emotional violence?**
Emotional violence includes, for example, threats, insults, controlling of social contacts.
(0) Never (1) Occasionally (2) Often

5. **Has your current partner ever subjected you to sexual violence?**
Sexual violence includes, for example, sexual humiliation, assault, rape.
(0) Never (1) Occasionally (2) Often

6. **Are you currently seeking medical care for an injury caused by your *current* partner?**
(0) Yes (1) No (2) I prefer not to answer

7. **Are you currently seeking medical care for an injury caused by violence from another person?**
(0) Yes (1) No (2) I prefer not to answer

8. **If you answered yes, what is your relationship with the person who committed the violence?**

9. **Was the person who committed the violence under the influence of any substance when they hurt you?**
(0) Yes, alcohol (1) Yes, drugs (2) I do not know (3) No

10. **Have you previously been seeking medical care for an injury caused by violence in a close relationship?**
(0) Yes: (*circle this if it happened in the current/past relationship*) (1) No (2) I prefer not to answer

11. **Do you think it is important that health care services ask about violence in close relationships?**
(0) Yes (1) No

12. **What is your level of education?**
(0) Primary school (1) High School (2) College/University

13. If you have been subjected to violence in a **current** close relationship, you have the option to receive counselling with a social worker at the trauma center at Mölndal Hospital. You will be called for a return visit in 1-2 weeks and the social worker's visit will not be visible in your medical record or on the call on paper you receive at home. The call will look like a regular call for a medical appointment. If your injury also requires a medical follow-up, you will be scheduled to see the social worker after your medical appointment. The social worker is subject to existing healthcare laws. Do you wish to speak to a social worker?

For peer review only

DORIS

Answer sheet A
please check the option that best applies to you

---

1. [0] Less than 1 year [1] 1-5 years [2] 6-10 years [3] More than 10 years [4] I have no partner

If you have answered ***that you do not have a partner*** *you can skip to question 7!*

2. [0] Man [1] Woman [2] I prefer not to answer

3. [0] Never [1] Occasionally [2] Often

4. [0] Never [1] Occasionally [2] Often

5. [0] Never [1] Occasionally [2] Often

6. [0] Yes [1] No [2] I prefer not to answer

7. [0] Yes [1] No [2] I prefer not to answer

8. ____

9. [0] Yes, alcohol [1] Yes, drugs [2] I do not know [3] No

10. [0] Yes: currently / in the past [1] No [2] I prefer not to answer

If you have answered yes, how many times have you been seeking medical care: ___________

11. [0] Yes [1] No

12. [0] Primary school [1] High School [2] College/University

13. [0] Yes [1] No

Consent to the DORIS study
I have been provided with the written information and hereby consent to the processing of my information in the DORIS study as described in the information for research participants.

______________________ ______________________
Signature Clarification of signature

______________________ ______________________
National identification number Date

For peer review only

DORIS

Questionnaire B

What we mean by violence in close relationships is the emotional, physical or sexual violence that someone you feel emotionally close to (in the household, family or partner relationship) has subjected you to.

1. Are you currently seeking medical care for an injury caused by violence in a close relationship?

YES NO

2. Do you think that health care services should routinely ask about violence in close relationships as the cause of injuries?

YES NO

For peer review only

DORIS

Answer sheet B
please circle the answer that best applies to you

1. YES NO

2. YES NO

For peer review only

DORIS

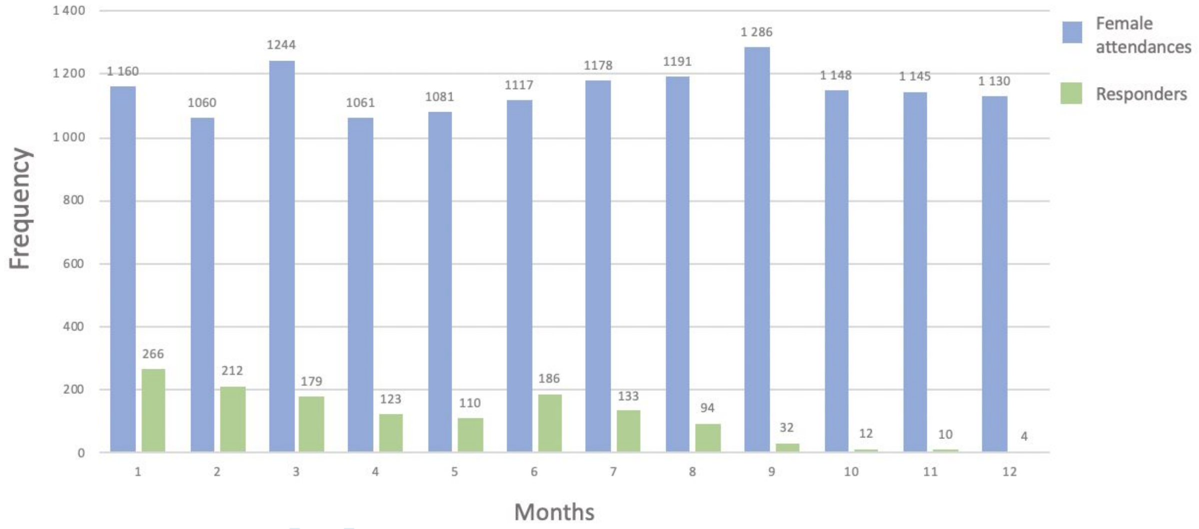

Supplementary material, Figure S1. The responder frequency per month The number of unique female attendances (blue) and responders (green) per study month (21st to 21st of the next calendar month).

For peer review only

DORIS

The incidence of DOmestic violence in ORthopaedIcS as a cause of acute injury in female patients

Karin Svensson Malchau MD PhD[1], Eva-Corina Caragounis MD PhD[2], Mikael Sundfeldt MD PhD[1]

[1]Department of Orthopaedics, Institute of Clinical Sciences, The Sahlgrenska Academy, Gothenburg University, Gothenburg, Sweden
[2]Department of Surgery, Institute of Clinical Sciences, The Sahlgrenska Academy, Gothenburg University, Gothenburg, Sweden

Background

Intimate partner violence (IPV) is defined as abuse within a close partner relationship and can be both psychological, sexual and/or physical. According to the Centres for Disease Control and Prevention (CDC), one in four women and one in ten men experience IPV in the United States.[1] The corresponding numbers in Sweden are one in four women and one in six men, and in a nationwide study from 2012 the prevalence of IPV was 7.0%.[2] The same study concluded a life-time prevalence of physical abuse in a former or current relationship of 15% in women.[2] Reportedly, IPV leads to physical injuries in 19-55% of women who have been made subject to it.[3] Victims of IPV are at a greater risk for physical and mental health issues,[3] and IPV has economic consequences such as medical costs, absence from work and legal costs alongside the individual's suffering.[4] Further, IPV can lead to death and nearly 20% of female homicides, in which the relationship to the perpetrator is known, are caused by a former or current intimate partner.[5, 6] IPV is also correlated with a greater risk of suicide.[3, 7, 8]

IPV has been described as a step-wise process starting off with phases of verbal and systematic psychological abuse and thereafter often leading to physical abuse.[7] IPV is one of the most common cause for injuries in women. Several studies have evaluated which injuries are typical in patients with IPV and musculoskeletal injuries are one of the most common presentations.[9] An international multi-centre study investigating the prevalence in orthopaedic clinics concluded that one in 50 women present with an injury directly due to IPV.[10] In an orthopaedic setting, fractures are the most frequent injury due to IPV followed by dislocations or sprains of joints.[11] The prevalence of IPV in female patients at a fracture clinic has been reported at 32%.[12] Sprague *et al.* concluded that the recognition of IPV as a mechanism of injury is important, and orthopaedic clinics have previously been suggested as a good setting in which to screen for IPV.[11]

Since IPV can result in injuries needing medical attention, medical workers play an important role in the identification of victims. However, nearly one third of victims report that they are not offered the help or support they need when they contact authorities.[2] It can be difficult to detect IPV in a busy clinical setting such as the emergency room (ER), and staff may be unaware of which injuries are related to IPV. Around 75% of female patients think that healthcare staff, and specifically orthopaedic surgeons, should ask about IPV.[12] In one study, female patients reported that no orthopaedic surgeon asked them if IPV was a cause of their injury,[10] and few orthopaedic surgeons report screening injured patients for IPV.[14] When asked, orthopaedic surgeons under-estimated the prevalence, and a majority (80%) of surgeons believed that IPV occurred in less than 1% of their patients.[13] This highlights the importance to raise awareness of this cause of injury.

For peer review only

DORIS

In Sweden, only 29% of women who have been severely physically abused seek medical attention.[2] Bhandari *et al.* found that 36% of women had been prevented from seeking medical attention.[9] A further challenge in capturing IPV cases may be that the patient does not disclose occurrence of abuse when asked. Abused women are more uncomfortable answering questions about abuse than non-abused women.[15] Several different screening instruments for IPV have been developed and adapted for use in clinical settings. However, direct questioning has proven most efficient in an orthopaedic setting.[11]

Research within IPV in orthopaedics in Sweden is scarce and little is known about the prevalence and how support programs best should be implemented in an orthopaedic clinic. Screening is not used in clinical routine, but the implementation of screening within a healthcare setting may lead to a greater detection of IPV. However, a greater detection alone may not lead to an increased rate of referral to specialist care or a decrease in abuse.

Surgeons have previously reported that they feel uncomfortable asking about IPV and that they have limited knowledge in what to do if their patient is a victim.[14] The current project aims to identify the prevalence of orthopaedic injuries caused by IPV in female patients at Mölndal's hospital and the regional trauma centre at Sahlgrenska University Hospital. A further aim is to establish whether there is a correlation between IPV and certain types of orthopaedic injuries. Within this project, a support program offered to victims of IPV at the orthopaedic clinic will be developed and the staff will be educated in IPV. The patient's perception of the support program will be evaluated.

Research questions

- What is the prevalence of orthopaedic injuries as a direct result of IPV in female patients?
- Do female patients who have been made subject to IPV present with a certain type of orthopaedic fracture/injury?
- How do female patients who have been made subject to IPV perceive the support they receive from a novel support program set up within healthcare?

Methods and materials

In this project, female patients with orthopaedic injuries who come to the ER at Mölndal's hospital will be approached with a questionnaire in which direct questioning will be used.[11] At the ER at Mölndal's hospital, patients are directed to an examination room one-by-one. The questionnaire will be handed to the patient by the health care staff at the ER whilst the patient is waiting for the physician. The staff will briefly explain the ongoing study and the patient will be able to go through the study information and questionnaire privately. Upon study enrolment, written consent will be obtained.

If a patient reports on IPV she will be offered an out-patient appointment to the orthopaedic clinic where she will have the opportunity to talk in-depth with a welfare officer. The summoning letter to this out-patient visit is sent by mail. The letter will not mention the appointment with the welfare officer and will merely summon to a control of the fracture. This is intentional in order to protect the patient in cases where the patient's partner is a cohabitant.

For peer review only

DORIS

Once the patient returns to her out-patient appointment she will meet a welfare officer and receive further help as a part of the new support program. Patients who have participated in the support program will be asked to complete an evaluation questionnaire of their experiences of study participation.

Study population

The inclusion criteria in this project are:

- The patient is female
- The patient is 18 years or older
- The patient presents with an orthopaedic injury at the emergency department at Mölndal's hospital or at the trauma ward at the Sahlgrenska University Hospital

The exclusion criteria in this project are:

- The patient is unable to provide consent due to any reason
- The patient is cognitively impaired

Study definitions

In this project IPV is defined as any type of psychological, emotional or physical abuse.

Orthopaedic injuries are defined as fractures of the extremities, joint dislocations, joint strains or trauma to the extremities resulting in pain severe enough for medical attention.

An intimate partner relationship is defined as a partnership lasting at least one month.

Statistical analysis

The results of the questionnaires will be inputted manually in a database by the research group. The prevalence of orthopaedic injuries in female patients caused by IPV will be presented using descriptive statistics. A multivariable regression analysis will be conducted to investigate selected demographic characteristics including type of injury and their association to IPV. The project evaluation survey will be presented using descriptive statistics.

Data will be analysed using SPSS Statistics (version 26, IBM corporation, USA).

Clinical impact

Identifying the prevalence of orthopaedic fractures caused by IPV is of great importance to establish the magnitude, and increase awareness, of this problem. The identification of IPV may lead to interventions from the health care staff which in turn may increase the patient's security and health. Identifying IPV may even lead to life-saving interventions. Further, it is of importance to acquire a greater understanding for what support model is desired by patients who present to an orthopaedic clinic with injuries due to IPV to improve the care of these patients.

For peer review only

DORIS

Study group
Mikael Sundfeldt (MS), Karin Svensson Malchau (KSM) and Eva-Corina Caragounis (ECC) have designed the study and intend on involving a welfare officer and research nurse part-time within the project. The research nurse will collect the questionnaires from the ER and KSM/MS will create and input data in a database. The research nurse will coordinate scheduled visits to the welfare officer. The welfare officer will help the patients in need of further support.

Time plan
Study start is planned for June 2021 and the study will continue for one year onward to June 2022. Data will continuously be inputted in the database. Data will be analysed and the manuscript will be prepared between July to October 2022.

References

1. Smith SG, Zhang, X., Basile, K.C., Merrick, M.T., Wang, J., Kresnow, M., Chen, J. The National Intimate Partner and Sexual Violence Survey (NISVS): 2015 Data Brief - Updated Release 2018 [Available from: https://www.cdc.gov/violenceprevention/pdf/2015data-brief508.pdf.
2. BRÅ. Brott i nära relationer 2014 [Available from: https://www.bra.se/download/18.9eaaede145606cc8651ff/1399015861526/2014_8_Brott_i_nara_relationer.pdf.
3. Ellsberg M, Jansen HA, Heise L, Watts CH, Garcia-Moreno C, Health WHOM-cSoWs, et al. Intimate partner violence and women's physical and mental health in the WHO multi-country study on women's health and domestic violence: an observational study. Lancet. 2008;371(9619):1165-72.
4. Max W, Rice DP, Finkelstein E, Bardwell RA, Leadbetter S. The economic toll of intimate partner violence against women in the United States. Violence Vict. 2004;19(3):259-72.
5. Centres for Disease Control and Prevention 2020 [Available from: https://www.cdc.gov/violenceprevention/intimatepartnerviolence/fastfact.html.
6. Jack SPD, Petrosky E, Lyons BH, Blair JM, Ertl AM, Sheats KJ, et al. Surveillance for Violent Deaths - National Violent Death Reporting System, 27 States, 2015. MMWR Surveill Summ. 2018;67(11):1-32.
7. SKR. En kunskapsöversikt: mäns vål mot kvinnor i nära relationer. 2006.
8. Dufort M, Stenbacka M, Gumpert CH. Physical domestic violence exposure is highly associated with suicidal attempts in both women and men. Results from the national public health survey in Sweden. Eur J Public Health. 2015;25(3):413-8.
9. Bhandari M, Dosanjh S, Tornetta P, 3rd, Matthews D, Violence Against Women Health Research C. Musculoskeletal manifestations of physical abuse after intimate partner violence. J Trauma. 2006;61(6):1473-9.
10. Investigators P, Sprague S, Bhandari M, Della Rocca GJ, Goslings JC, Poolman RW, et al. Prevalence of abuse and intimate partner violence surgical evaluation (PRAISE) in orthopaedic fracture clinics: a multinational prevalence study. Lancet. 2013;382(9895):866-76.

For peer review only

11. Sprague S, Madden K, Dosanjh S, Petrisor B, Schemitsch EH, Bhandari M. Screening for intimate partner violence in orthopedic patients: a comparison of three screening tools. J Interpers Violence. 2012;27(5):881-98.
12. Bhandari M, Sprague S, Dosanjh S, Petrisor B, Resendes S, Madden K, et al. The prevalence of intimate partner violence across orthopaedic fracture clinics in Ontario. J Bone Joint Surg Am. 2011;93(2):132-41.
13. Bhandari M, Sprague S, Tornetta P, 3rd, D'Aurora V, Schemitsch E, Shearer H, et al. (Mis)perceptions about intimate partner violence in women presenting for orthopaedic care: a survey of Canadian orthopaedic surgeons. J Bone Joint Surg Am. 2008;90(7):1590-7.
14. Della Rocca GJ, Sprague S, Dosanjh S, Schemitsch EH, Bhandari M. Orthopaedic surgeons' knowledge and misconceptions in the identification of intimate partner violence against women. Clin Orthop Relat Res. 2013;471(4):1074-80.
15. Brown JB, Lent B, Schmidt G, Sas G. Application of the Woman Abuse Screening Tool (WAST) and WAST-short in the family practice setting. J Fam Pract. 2000;49(10):896-903.

For peer review only

STROBE Statement—Checklist of items that should be included in reports of ***cohort studies***

	Item No	Recommendation	Page No
**Title and abstract**	1	(*a*) Indicate the study's design with a commonly used term in the title or the abstract<br>(*b*) Provide in the abstract an informative and balanced summary of what was done and what was found	1
**Introduction**			
Background/rationale	2	Explain the scientific background and rationale for the investigation being reported	4
Objectives	3	State specific objectives, including any prespecified hypotheses	4, 6
**Methods**			
Study design	4	Present key elements of study design early in the paper	5
Setting	5	Describe the setting, locations, and relevant dates, including periods of recruitment, exposure, follow-up, and data collection	5
Participants	6	(*a*) Give the eligibility criteria, and the sources and methods of selection of participants. Describe methods of follow-up<br>(*b*) For matched studies, give matching criteria and number of exposed and unexposed	6
Variables	7	Clearly define all outcomes, exposures, predictors, potential confounders, and effect modifiers. Give diagnostic criteria, if applicable	6
Data sources/ measurement	8*	For each variable of interest, give sources of data and details of methods of assessment (measurement). Describe comparability of assessment methods if there is more than one group	6
Bias	9	Describe any efforts to address potential sources of bias	6
Study size	10	Explain how the study size was arrived at	-
Quantitative variables	11	Explain how quantitative variables were handled in the analyses. If applicable, describe which groupings were chosen and why	6
Statistical methods	12	(*a*) Describe all statistical methods, including those used to control for confounding<br>(*b*) Describe any methods used to examine subgroups and interactions<br>(*c*) Explain how missing data were addressed<br>(*d*) If applicable, explain how loss to follow-up was addressed<br>(*e*) Describe any sensitivity analyses	6
**Results**			
Participants	13*	(a) Report numbers of individuals at each stage of study—eg numbers potentially eligible, examined for eligibility, confirmed eligible, included in the study, completing follow-up, and analysed<br>(b) Give reasons for non-participation at each stage<br>(c) Consider use of a flow diagram	7
Descriptive data	14*	(a) Give characteristics of study participants (eg demographic, clinical, social) and information on exposures and potential confounders<br>(b) Indicate number of participants with missing data for each variable of interest<br>(c) Summarise follow-up time (eg, average and total amount)	7
Outcome data	15*	Report numbers of outcome events or summary measures over time	7

For peer review only

For peer review only - http://bmjopen.bmj.com/site/about/guidelines.xhtml

Main results	16	(*a*) Give unadjusted estimates and, if applicable, confounder-adjusted estimates and their precision (eg, 95% confidence interval). Make clear which confounders were adjusted for and why they were included<br>(*b*) Report category boundaries when continuous variables were categorized<br>(*c*) If relevant, consider translating estimates of relative risk into absolute risk for a meaningful time period	-
Other analyses	17	Report other analyses done—eg analyses of subgroups and interactions, and sensitivity analyses	-
**Discussion**			
Key results	18	Summarise key results with reference to study objectives	9
Limitations	19	Discuss limitations of the study, taking into account sources of potential bias or imprecision. Discuss both direction and magnitude of any potential bias	9-12
Interpretation	20	Give a cautious overall interpretation of results considering objectives, limitations, multiplicity of analyses, results from similar studies, and other relevant evidence	13
Generalisability	21	Discuss the generalisability (external validity) of the study results	13
**Other information**			
Funding	22	Give the source of funding and the role of the funders for the present study and, if applicable, for the original study on which the present article is based	14

*Give information separately for exposed and unexposed groups.

Note: An Explanation and Elaboration article discusses each checklist item and gives methodological background and published examples of transparent reporting. The STROBE checklist is best used in conjunction with this article (freely available on the Web sites of PLoS Medicine at http://www.plosmedicine.org/, Annals of Internal Medicine at http://www.annals.org/, and Epidemiology at http://www.epidem.com/). Information on the STROBE Initiative is available at http://www.strobe-statement.org.

For peer review only

BMJ Open

The DORIS study: Domestic violence in ORthopaedIcS, a prospective cohort study on the annual prevalence of domestic violence in orthopaedic emergency care

Journal:	*BMJ Open*
Manuscript ID	bmjopen-2024-085618.R1
Article Type:	Original research
Date Submitted by the Author:	04-Apr-2024
Complete List of Authors:	Svensson Malchau, Karin; Sahlgrenska University Hospital, Orthopaedics<br>Caragounis, Eva-Corina; University of Gothenburg, Department of Surgery, Institute of Clinical Sciences<br>Sundfeldt, Mikael; Sahlgrenska University Hospital, Orthopaedics
**Primary Subject Heading**:	Epidemiology
Secondary Subject Heading:	Public health, Emergency medicine, Patient-centred medicine
Keywords:	ACCIDENT & EMERGENCY MEDICINE, Awareness, Health, Mass Screening, ORTHOPAEDIC & TRAUMA SURGERY

SCHOLARONE™
Manuscripts

I, the Submitting Author has the right to grant and does grant on behalf of all authors of the Work (as defined in the below author licence), an exclusive licence and/or a non-exclusive licence for contributions from authors who are: i) UK Crown employees; ii) where BMJ has agreed a CC-BY licence shall apply, and/or iii) in accordance with the terms applicable for US Federal Government officers or employees acting as part of their official duties; on a worldwide, perpetual, irrevocable, royalty-free basis to BMJ Publishing Group Ltd ("BMJ") its licensees and where the relevant Journal is co-owned by BMJ to the co-owners of the Journal, to publish the Work in this journal and any other BMJ products and to exploit all rights, as set out in our licence.

The Submitting Author accepts and understands that any supply made under these terms is made by BMJ to the Submitting Author unless you are acting as an employee on behalf of your employer or a postgraduate student of an affiliated institution which is paying any applicable article publishing charge ("APC") for Open Access articles. Where the Submitting Author wishes to make the Work available on an Open Access basis (and intends to pay the relevant APC), the terms of reuse of such Open Access shall be governed by a Creative Commons licence – details of these licences and which Creative Commons licence will apply to this Work are set out in our licence referred to above.

Other than as permitted in any relevant BMJ Author's Self Archiving Policies, I confirm this Work has not been accepted for publication elsewhere, is not being considered for publication elsewhere and does not duplicate material already published. I confirm all authors consent to publication of this Work and authorise the granting of this licence.

For peer review only

DORIS

The DORIS study: Domestic violence in ORthopaedIcS, a prospective cohort study on the annual prevalence of domestic violence in orthopaedic emergency care

Karin Svensson Malchau MD PhD[1], Eva-Corina Caragounis MD PhD[2], Mikael Sundfeldt MD PhD[1]

[1]Department of Orthopaedics, Institute of Clinical Sciences, Sahlgrenska University Hospital, Sahlgrenska Academy, University of Gothenburg, Gothenburg, Sweden
[2]Department of Surgery, Institute of Clinical Sciences, Sahlgrenska University Hospital, Sahlgrenska Academy, University of Gothenburg, Gothenburg, Sweden

Corresponding author: Karin Svensson Malchau, Department of Orthopaedics, R-huset, Länsmansvägen 28, 431 42 Mölndal, Sweden. E-mail address: karin.am.svensson@vgregion.se, telephone number +46(0)313430761.

For peer review only

ABSTRACT

Background

Domestic violence (DV) is a major problem which despite many efforts persists globally. Victims of DV can present with various injuries, whereof musculoskeletal presentation is common.

Objectives

The DORIS study aimed to establish the annual prevalence of DV at an orthopaedic emergency department (ED) in Sweden.

Design

Female adult patients with orthopaedic injuries seeking treatment at a tertiary orthopaedic centre between September 2021–2022 were screened during their ED visit.

Setting

A single-centre study at a tertiary hospital.

Participants

Adult female patients seeking care for acute orthopaedic injuries were eligible for the study. During the study period, 4,192 female patients were provided with study forms and 1,366 responded (32.5%).

Primary and secondary outcome measures

The primary outcome measure was to establish the annual prevalence of injuries due to DV and secondly, establish the rate of current experience of any type of DV.

Results

One in 14 had experience of current DV (n=100, 7.5%) and one in 65 (n=21, 1.5%) had an injury due to DV.

Conclusions

The prevalence of DV found in the current study is comparable to international findings and adds to the growing body of evidence that it needs to be considered in clinical practice. It is important to raise awareness of DV, and frame strategies, as healthcare staff have a unique position to identify and offer intervention to DV victims.

ARTICLE SUMMARY

Strengths and limitations of this study

- This is a prospective observational study investigating the annual prevalence of domestic violence (DV) in female orthopaedic patients using questionnaires containing validated questions for DV.
- Study participants were approached individually without the presence of company and great discretion was taken to ascertain the safety of DV victims.
- The study was designed to screen all female patients consecutively, and although difficulties in the practical implementation of the screening program impeded the desired inclusion rate, a large volume of patients were included.
- Study participants could not choose to be anonymous which may have deterred some patients from filling out the study questionnaires.

Keywords: orthopaedics, domestic violence, trauma

Introduction

Domestic violence (DV) is a serious public health problem estimated to affect as many as 27% of women in partner relationships during their lifetime.[1] It is an insidious process, starting off with phases of systematic psychological abuse often leading to physical abuse.[2] Aside its societal and individual economic consequences,[3] it is one of the most common causes for physical injuries in women and victims are at great risk for mental health issues,

For peer review only - http://bmjopen.bmj.com/site/about/guidelines.xhtml

DORIS

suicide and homicide.[4] 20–50% of female homicides, are caused by a former or current intimate partner,[5, 6] and in Sweden, the death toll due to known DV was 13 in 2020.[7]

Musculoskeletal injuries are one of the most common presentations of DV.[8, 9] One in 50 women present to fracture clinics with an injury due to DV.[10] Recognition of DV as an injury mechanism is important and orthopaedic units have been suggested ideal for screening.[11, 12] However, the difficulties of identifying DV are many. Victims may be prevented from seeking medical attention by their abuser which was found true for 36% of women in Canada.[8] A further challenge is the absence of active questioning in healthcare and that patients may not disclose occurrence of abuse.[13] Orthopaedic surgeons under-estimate the prevalence of DV,[14] and do not ask about DV.[10]

Implementation of screening within healthcare may lead to a greater detection of DV, which in turn can be potentially lifesaving. Nevertheless, questioning for DV is not standard and formal documentation is poor.[15] Sweden is considered the most gender equal country in the European Union,[7] however, research on DV in orthopaedics is scarce and little is known about its prevalence in Sweden. The current project aimed to identify the annual prevalence of orthopaedic injuries caused by DV and current experience of DV, in female patients at the largest orthopaedic emergency department (ED) in Sweden. Types of DV, injury due to DV and stated injury mechanisms were also evaluated.

Methods

Study design

This is a self-reported questionnaire-based study including questions validated for detection of partner violence in an orthopaedic setting.[11]

For peer review only - http://bmjopen.bmj.com/site/about/guidelines.xhtml

Objectives

The primary objective was to identify the annual prevalence of orthopaedic injuries sustained directly due to DV. The secondary objectives were to establish the annual prevalence of current experience of DV and investigate which types of DV, injuries and stated injury mechanisms were most common.

Setting

The study was conducted at the ED of the Sahlgrenska University Hospital/Mölndal in Gothenburg, Sweden from 21st September 2021 to 21st September 2022. The ED averages 45,000 unique attendances yearly and the orthopaedic section has an average of 38 female attendances daily.

Sets of study information, marked with name and social security number, were assembled upon triage. Staff were instructed to hand out the forms to all female patients fulfilling the study inclusion criteria. Forms were handed out in the examination room, filled out in private, and put in a sealed envelope (Figure 1). ED staff were unaware of status of study participation. The forms were contained inside the ED as a precautious measure to diminish the risk of unauthorized persons identifying potential victims. If ED staff discovered a case of DV when informing patients about the study, they were asked to mark the envelope with an “X”. However, the patient was only included in the further analysis if she consented to study participation. Medical records of consenting patients reporting DV were reviewed to assess injury type and severity.

For peer review only

For peer review only - http://bmjopen.bmj.com/site/about/guidelines.xhtml

DORIS

Patients who wished to meet a project counsellor were booked for a medical follow-up without mention of the counsellor. This was intentional to protect the patient in cases of cohabitation with the abuser.

Participants

Patients of female sex of at least 18 years of age and with residency in Sweden triaged to the orthopaedic section of the ED were included in the study. Patients accompanied by someone, or with cognitive impairment or physical impairment, i.e. dementia or poor eyesight, were excluded. Furthermore, patients who could not understand Swedish, English or Arabic were also excluded. No sample size calculation was conducted as the objective was to establish the annual prevalence of DV victims.

Study questionnaire

Screening was performed using paper questionnaires, which had been developed based on the work of Sprague et al., where the direct questioning approach detected DV to a greater extent than other tools evaluated for orthopaedic use .[11] Additional questions on demography were added (see Supplementary material). There were two forms (A and B) of which B was simplified and more anonymous in order to encourage higher responder rates (Supplementary material, S1). Participants received both forms and could choose which form to fill in. Study forms were provided in Swedish and translated two-way in English and Arabic.

Definitions

DV was defined as emotional, physical or sexual abuse. Any occurrence within the family, domestic unit or by former intimate partners, was included, as defined by the Istanbul Convention (2011).[16] A relationship was defined as a partnership lasting at least one month.

For peer review only - http://bmjopen.bmj.com/site/about/guidelines.xhtml

DORIS

Data analysis

	**All responders** (n=1,361)	**Responders reporting no experience of DV in current relationship** (n=1,165)	**Responders reporting current DV, not DV as direct cause of injury** (n=79)	**Responders reporting DV as direct cause of injury** (n=16)	**Responders with missing or incomplete answers on current DV** (n=101)
**Age** (range, years)					
18-29	226 (16.6)	201 (17.3)	8 (10.1)	2 (12.5)	15 (14.8)
30-39	211 (15.5)	182 (15.6)	13 (16.5)	4 (25.0)	12 (11.9)
40-49	211 (15.5)	174 (14.9)	17 (21.5)	4 (25.0)	16 (15.8)
50-59	262 (19.3)	234 (20.1)	15 (19.0)	1 (6.3)	12 (11.9)
60-69	239 (17.6)	199 (17.1)	17 (21.5)	4 (25.0)	19 (18.8)
>70	202 (14.8)	167 (14.3)	8 (10.1)	1 (6.3)	26 (25.7)
Missing	10 (0.7)	8 (0.7)	1 (1.3)	0 (0.0)	1 (1.0)

Data was analysed descriptively with frequency counts and percentages for categorical variables. Software IBM SPSS version 29 was used for data analysis.

Ethical considerations

Written consent was obtained upon study enrolment in line with the Ethical Review Board's regulations (DNR 2021-01752).

Patient and Public Involvement

It was not deemed appropriate to involve patients or the public in the design, or conduct, or reporting, or dissemination plans of our research.

Results

In total, 4,192 (30.4%) out of 13,801 unique female attendances registered at the orthopaedic section of the ED were given study forms. Of these, 1,366 (32.6%) agreed to inclusion (Figure 2). The majority of responders spoke Swedish (99.4%), did not live in a socially disadvantaged area (80.4%) and were in a relationship (62.2%) (Table 1).

For peer review only

DORIS

**Resident of a socially disadvantaged area**					
Yes	227 (16.7)	184 (15.8)	16 (20.3)	3 (18.8)	24 (23.8)
No	1101 (80.9)	954 (81.9)	58 (73.4)	13 (81.3)	75 (75.2)
Protected person/not a resident in Gothenburg	9 (0.7)	6 (0.1)	3 (3.8)	0 (0.0)	0 (0.0)
Missing	24 (1.8)	21 (1.8)	2 (2.5)	0 (0.0)	1 (1.0)
**Language**					
Swedish	1353 (99.4)	1161 (99.7)	77 (97.5)	15 (94.1)	100 (99.0)
English	5 (0.4)	3 (0.3)	1 (1.3)	0 (0.0)	1 (1.0)
Arabic	3 (0.2)	1 (0.0)	1 (1.3)	1 (5.9)	0 (0.0)
**Education level**					
Compulsory school	104 (7.6)	82 (7.0)	5 (6.3)	2 (12.5)	15 (14.9)
High school	459 (33.7)	395 (33.9)	30 (38.0)	9 (56.3)	25 (24.8)
University	727 (53.4)	650 (55.8)	43 (54.4)	4 (25.0)	29 (28.7)
Missing	71 (5.3)	38 (3.3)	1 (1.3)	1 (6.3)	31 (30.7)
**Partner sex**					
No partner	430 (31.6)	427 (36.7)	0 (0.0)	3 (17.6)	0 (0.0)
Male	806 (59.2)	711 (61.0)	75 (95.0)	10 (64.7)	10 (10.0)
Female	40 (2.9)	22 (2.0)	2 (2.5)	2 (11.8)	14 (13.9)
Missing	85 (6.2)	5 (0.4)	2 (2.5)	1 (5.9)	77 (76.2)
**Duration of relationship**					
Less than one year	36 (2.6)	30 (2.6)	4 (5.1)	2 (12.5)	1 (1.0)
1-5years	165 (12.1)	144 (12.4)	12 (15.2)	3 (18.8)	6 (5.9)
6-10 years	96 (7.1)	80 (6.9)	12 (15.2)	2 (12.5)	2 (2.0)
More than 10 years	567 (41.7)	476 (40.9)	50 (63.3)	5 (31.3)	36 (35.6)
No partner	440 (32.3)	430 (36.9)	1 (1.3)	3 (18.8)	6 (5.9
Missing	57 (4.2)	5 (0.4)	0 (0.0)	2 (12.5)	50 (50.0)
**Have you ever sought medical care for DV?**					
No	1050 (77.1)	942 (80.9)	57 (72.2)	8 (50.0)	43 (42.6)
Yes	54 (4.0)	38 (3.3)	10 (12.7)	3 (18.8)	4 (4.0)
Missing	257 (18.9)	185 (15.9)	13 (16.5)	5 (31.3)	54 (53.5)
**Should health care workers ask about DV?**					
Yes	1209 (88.8)	1068 (91.7)	76 (96.2)	14 (87.5)	51 (50.5)
No	41 (3.0)	30 (2.6)	2 (2.5)	0 (0.0)	9 (8.9)
Missing	111 (8.2)	67 (5.8)	1 (1.3)	2 (12.5)	41 (40.6)

Table 1. Demographics of all responders and whether health care should ask about DV grouped by experience of DV.

Experience of DV

Of the 1,366 patients, 100 patients (7.5%) had current experience of DV and 21 (1.5%) of them had an injury due to DV. Of the 21 patients, 16 consented to filling out the study forms. The remaining five patients disclosed DV to healthcare staff but declined to fill out the study

For peer review only

DORIS

forms. Therefore, they were not included in the further analysis, leaving 95 patients of the 100 patients who had stated current experience of DV, eligible for further analysis (Figure 2).

DV (any type) was reported by 89 (89/1361, 6.5%) patients in their current relationship. Emotional abuse was most common (69/89, 77.5%) followed by physical abuse (33/89, 37.1%) and sexual abuse (19/89, 21.3%) (Figure 3).

	**Frequency** n (%)
**Formal documentation of DV in medical record**	
Yes	8 (50)
No	8 (50
**Stated injury mechanism in medical record**	
Fall trauma, unspecified	8 (50)
Abuse	8 (50)
**Orthopaedic treatment**	
Pain medication and physiotherapy	8 (50)
Immobilization (cast/orthosis)	6 (38)
Surgery	2 (12)
**Need for sick leave**	
Yes	5 (31)

DV as a direct cause of injury

In total, 21 patients with an injury due to DV were identified (Figure 2), meaning that one in 65 patients needed medical attention due to physical abuse. Of the 16 consenting DV victims, eight had previously been in contact with healthcare for an injury due to abuse. Formal documentation of DV was noted in eight medical records, and in the remaining cases the injury mechanism was unspecified fall trauma (Table 2).

For peer review only

Table 2. Injury mechanism as stated in the medical records and treatment needs due to DV

No	11 (69)

The age span of DV victims was 18–76 years. Three patients were from socially disadvantaged areas and three patients had female partners. The majority of patients had completed high school but had no further academic education (Table 1). Eight patients reported on repeated abuse in their current relationship of which five stated an occurrence of both emotional, physical, and sexual abuse.

Fractures were the most prevalent injury followed by contusions and joint distortions (Table 3). Five patients sustained injuries requiring sick leave and two patients required surgery (Table 3). Thirty-seven follow-up visits were recorded due to DV injuries (excluding visits to the counsellor).

For peer review only

DORIS

Table 3. Type of injuries noted in cases with DV as direct cause of injury

Injury type and localisation	Frequency n (%)
**Fracture**	6 (38)
Hand	5
Foot	1
**Contusion**	4 (25)
Upper extremity	1
Lower extremity	3
**Distortion**	4 (25)
Shoulder	1
Knee	1
Foot	2
**Joint dislocation**	1 (6)
**Ligament rupture**	1 (6)
**Laterality of injury**	
Right	10 (63)
Left	4 (25)
Missing	2 (12)

Screening for DV

In total, 1,208 women (89.0%) were of the opinion that healthcare staff should ask about DV (Table 1). However, two of the 16 patients (12.5%) injured due to DV did not feel that screening was necessary. Fifty-four patients (4.0%) had previously contacted healthcare for physical abuse (Table 1), whereof 34 of these patients were still in an abusive relationship.

For peer review only - http://bmjopen.bmj.com/site/about/guidelines.xhtml

The project counsellors had contact with 23 patients whereof 8 had been injured due to DV. 12 patients (52.2%) showed up for their appointment. Six patients failed to appear, four had misunderstood or were too injured to come for their appointment. One patient had given a faulty address and did not respond to phone calls.

Discussion

The DORIS study aimed to establish the prevalence of injuries directly caused by DV, current experience of DV, types of DV, injuries and stated injury mechanisms in female patients in the largest orthopedic ED in Northern Europe. It also evaluated the rate of types of DV, injuries due to DV and what injury mechanisms were stated by victims. A rate of one in 14 patients (100/1366, 7.5%) with current experience of DV and one in 65 patients (21/1366, 1.6%) injured due to DV was established.

The prevalence of injuries due to DV (1.6%) is within the span of prevalence reported by the PRAISE group (0-3%), who conducted a multi-national investigation of intimate partner violence in female patients at orthopaedic injury clinics.[10] Current experience of DV was recorded in the DORIS study whereas previous studies have investigated the 12-month prevalence. A 12-month prevalence of DV of 15–22% in orthopaedic patients has previously been reported.[10, 17] In the DORIS study, 6.5% (89/1366) experienced DV in a current partner relationship. Differences in recruitment methods, study settings and staff engagement could serve as explanations to the lower prevalence in Sweden. The lower prevalence may also reflect governmental and societal policies on gender equality in Sweden.

For peer review only

For peer review only - http://bmjopen.bmj.com/site/about/guidelines.xhtml

DORIS

When comparing proportions of type of abuse, the present study established that emotional abuse was most common. This is also true in Scottish, American and Canadian settings.[10, 17] However, surprisingly, in the Netherlands and in Denmark, countries seemingly more comparable to Sweden, physical abuse was most common.[10] It may be difficult to understand what is meant by emotional abuse, the DORIS study forms contained examples of emotional abuse which may explain the higher prevalence.

Formal documentation of DV was noted in 50% of cases, meaning that 50% were not identified in the regular healthcare setting. Routine screening of DV leads to higher detection rates,[18] however, only 2% of healthcare workers in orthopedics routinely ask about it.[19] Surgeons feel uncomfortable and unsure of what to do if their patient is a victim which calls for better education and support models within healthcare.[20, 21]

Although it is important to be suspicious of inconsistent injury mechanisms or “red flags”, such as falling down the stairs,[22] feasible injury mechanisms were disclosed in 50% of the DV cases. Hence, questioning for DV should not just be conducted when suspicion is raised, as is often the case. Within the DORIS study, direct questioning, in questionnaire format, was used as this has proven efficient for DV screening and is less time consuming in an ED setting.[11, 23] However, the study forms contained a lot of text due to regulations stated by the Ethical Review Board, which may have discouraged potential responders. In the continued work of improving DV detection at the study site efforts will be made to optimise the screening tool.

Merely 50% of patients with an injury due to DV had previously been in contact with healthcare for DV. Hence, the remaining patients may have presented with an index injury. This finding supports the, previously suggested,[11, 19] need for screening in orthopaedic

For peer review only

settings, as early intervention can be potentially lifesaving. Up to 81% of female patients are of the opinion that healthcare staff should ask about DV.[10, 19, 23] The corresponding numbers were somewhat higher in the DORIS study (89% in the entire cohort and 94-96% in abused patients). Cultural differences and thereby expectations on healthcare may explain the aforementioned variances.

The strength of the DORIS study is its setting at the largest orthopaedic ED in Northern Europe. After, the PRAISE study,[10] DORIS is the largest prevalence study in orthopedics. Due to COVID restrictions during the study period, company was generally not allowed in the ED which facilitated the distribution of study forms. Victims of DV were also offered follow-up with a counsellor within the study.

A major limitation may be nonresponse bias. Although the study was regarded important by ED staff, the distribution rate of study forms was 30% and response rate 33%. The authors had meetings with ED staff and two counsellors were recruited to provide an in-house support program to increase the likelihood for staff engagement.[19] Unfortunately, due to management issues, the staffing situation became more turbulent with several experienced nurses and assistant nurses choosing to resign throughout the year. The authors believe that the inconsistencies in staffing were the main reason for poor study enrolment (Supplementary material, Figure 1). In addition, despite being an excellent forum for DV screening,[18] in regard to the "open window phase" (in which victims may be more receptive and prone to seek help after abuse),[24] the ED as such is a busy and stressful place. In general, detecting DV may be difficult in such a setting: staff may be unaware of DV as a problem, and patients may feel uncomfortable confiding in ED staff. For this reason, it is crucial to structure EDs in a

For peer review only

manner where triage can be done in private, as also suggested by Ahmad et al.,[18] and where patients are unaccompanied in triage as standard routine.

Poor response rate was partly expected. Similar studies,[10, 17] have had different approaches to recruitment making it difficult to evaluate what an acceptable response rate is. Due to the delicate nature of the study, the authors had preferred that social security number and further personal details were omitted when consenting to the study. The need to do this may have deterred potential victims from disclosing DV. However, full disclosure of personal details was a requirement from the Ethical Review Board due to research regulations. Furthermore, the authors have reason to believe that the 2,325 patients who for some reason did not wish to participate in the study may not have received proper study information or been given a chance to fill out the study forms.

The exclusion criteria imply certain limitations. Elderly patients, either accompanied by caregivers or with the diagnosis of dementia, were not included. Despite the difficulties of capturing cases in this group, it is important to acknowledge their vulnerability and that both dementia and female sex are predictive of abuse.[25] Furthermore, the authors acknowledge that DV affects both female and male patients. Screening of females was chosen as female DV patients have a greater fracture risk, 83% of ED visits due to DV are female, and 50% of female homicides are due to DV.[12] However, the long-term goal for the DORIS project is to provide a healthcare program dedicated to DV patients regardless of sex.

The DORIS study focused on current abuse, whereas previous research, such as conducted by the PRAISE group and Sardinha et al. also investigated life-time abuse.[1, 10, 17] In hindsight, the inclusion of life-time abuse would have been interesting for comparative reasons. However,

For peer review only

For peer review only - http://bmjopen.bmj.com/site/about/guidelines.xhtml

DORIS

when designing the study, the authors decided that the patient's current situation was the most clinically relevant and therefore most important.

Despite its limitations, and a probable under-reporting of DV, the finding of one in 65 patients translates to one victim of DV injuries nearly every second day, and two to three patients with current experience of DV daily, at the study center. Interventions are essential to disrupt continued abuse and healthcare has an important role in the detection of DV.[9, 26] The experience generated by the present study suggests that screening is necessary in order to improve identification of DV cases and that patients expect healthcare to engage in detecting DV. The results from the DORIS study will be used to improve routines at the study site, and hopefully inspire to similar actions elsewhere.

Conclusion

The prevalence of DV established in the current study implies a high annual volume of DV victims at the study site. DV victims may come to an orthopaedic setting with an index injury and healthcare staff have an unique opportunity to intervene. The DORIS study adds to the growing body of evidence that DV needs attention in the healthcare setting. Increased awareness and actions to identify DV is imperative, and it is important to educate, engage and provide adequate conditions for healthcare staff to conduct screening. Future work should focus on implementing DV screening as a routine and provide a safe environment for DV victims in all healthcare disciplines.

Author contributions

For peer review only - http://bmjopen.bmj.com/site/about/guidelines.xhtml

KSM participated in the planning and design of the study, collection of patient data, analyzed the data, interpreted the data, drafted the manuscript, and critically revised the manuscript. ECC participated in the planning and design of the study and critically revised the manuscript. MS participated in the planning and design of the study and critically revised the manuscript.

Acknowledgements

The authors would like to thank Sandra Rosnell, Linda Stolpe, Stella Sundfeldt, Ann-Christin von Corswant, Anne Louise Gidestrand, the medical staff at the ED at Mölndal's Hospital and all the patients who chose to participate.

Funding

The study was funded by Doktor Felix Neuberghs stiftelse N/A, BGS forskningsstipendium N/A, Göteborgs Läkaresällskap N/A, SU-fonderna N/A and Konrad och Helfrid Johanssons stiftelse N/A.

Competing interest statement

All authors have completed the ICMJE uniform disclosure form at http://www.icmje.org/disclosure-of-interest/ and declare: no support from any organisation for the submitted work; no financial relationships with any organisations that might have an interest in the submitted work in the previous three years; no other relationships or activities that could appear to have influenced the submitted work

Ethical considerations

Written consent was obtained upon study enrolment in line with the Ethical Review Board's regulations (DNR 2021-01752).

For peer review only

For peer review only - http://bmjopen.bmj.com/site/about/guidelines.xhtml

DORIS

Data availability statement

Data supporting this study cannot be shared publicly in order to protect DV victims.

References

1. Sardinha L, Maheu-Giroux M, Stockl H, Meyer SR, Garcia-Moreno C. Global, regional, and national prevalence estimates of physical or sexual, or both, intimate partner violence against women in 2018. Lancet. 2022;399(10327):803-13.
2. Murphy CM, O'Leary KD. Psychological aggression predicts physical aggression in early marriage. J Consult Clin Psychol. 1989;57(5):579-82.
3. Max W, Rice DP, Finkelstein E, Bardwell RA, Leadbetter S. The economic toll of intimate partner violence against women in the United States. Violence Vict. 2004;19(3):259-72.
4. Ellsberg M, Jansen HA, Heise L, Watts CH, Garcia-Moreno C, Health WHOM-cSoWs, et al. Intimate partner violence and women's physical and mental health in the WHO multi-country study on women's health and domestic violence: an observational study. Lancet. 2008;371(9619):1165-72.
5. Jack SPD, Petrosky E, Lyons BH, Blair JM, Ertl AM, Sheats KJ, et al. Surveillance for Violent Deaths - National Violent Death Reporting System, 27 States, 2015. MMWR Surveill Summ. 2018;67(11):1-32.
6. Stockl H, Devries K, Rotstein A, Abrahams N, Campbell J, Watts C, et al. The global prevalence of intimate partner homicide: a systematic review. Lancet. 2013;382(9895):859-65.
7. [Available from: https://eige.europa.eu/gender-equality-index/2022/domain/violence/SE.
8. Bhandari M, Dosanjh S, Tornetta P, 3rd, Matthews D, Violence Against Women Health Research C. Musculoskeletal manifestations of physical abuse after intimate partner violence. J Trauma. 2006;61(6):1473-9.
9. Giordano V, Giordano C, Lopes IM, Pires RE, Godoy-Santos A, Giannoudis PV. Orthopaedic surgeons can play important role in identifying victims of domestic violence in the emergency department - narrative review of Brazilian literature. Medicine (Baltimore). 2022;101(50):e31461.
10. Investigators P, Sprague S, Bhandari M, Della Rocca GJ, Goslings JC, Poolman RW, et al. Prevalence of abuse and intimate partner violence surgical evaluation (PRAISE) in orthopaedic fracture clinics: a multinational prevalence study. Lancet. 2013;382(9895):866-76.
11. Sprague S, Madden K, Dosanjh S, Petrisor B, Schemitsch EH, Bhandari M. Screening for intimate partner violence in orthopedic patients: a comparison of three screening tools. J Interpers Violence. 2012;27(5):881-98.
12. Loder RT, Momper L. Demographics and Fracture Patterns of Patients Presenting to US Emergency Departments for Intimate Partner Violence. J Am Acad Orthop Surg Glob Res Rev. 2020;4(2).

For peer review only

DORIS

13. Brown JB, Lent B, Schmidt G, Sas G. Application of the Woman Abuse Screening Tool (WAST) and WAST-short in the family practice setting. J Fam Pract. 2000;49(10):896-903.
14. Bhandari M, Sprague S, Tornetta P, 3rd, D'Aurora V, Schemitsch E, Shearer H, et al. (Mis)perceptions about intimate partner violence in women presenting for orthopaedic care: a survey of Canadian orthopaedic surgeons. J Bone Joint Surg Am. 2008;90(7):1590-7.
15. Khurana B, Sing D, Gujrathi R, Keraliya A, Bay CP, Chen I, et al. Recognizing Isolated Ulnar Fractures as Potential Markers for Intimate Partner Violence. J Am Coll Radiol. 2021;18(8):1108-17.
16. Council of Europe Convention on preventing and combating violence against women and domestic violence. Istanbul; 2011.
17. Downie S, Kanya I, Madden K, Bhandari M, Jariwala AC. Intimate partner violence (IPV) in male and female orthopaedic trauma patients: a multicentre, cross-sectional prevalence study. BMJ Open. 2021;11(8):e046164.
18. Ahmad I, Ali PA, Rehman S, Talpur A, Dhingra K. Intimate partner violence screening in emergency department: a rapid review of the literature. J Clin Nurs. 2017;26(21-22):3271-85.
19. Downie S, Madden K, Bhandari M, Jariwala A. A prospective questionnaire-based study on staff awareness of intimate partner violence (IPV) in orthopaedic trauma patients. Surgeon. 2019;17(4):207-14.
20. Della Rocca GJ, Sprague S, Dosanjh S, Schemitsch EH, Bhandari M. Orthopaedic surgeons' knowledge and misconceptions in the identification of intimate partner violence against women. Clin Orthop Relat Res. 2013;471(4):1074-80.
21. Sprague S, Swinton M, Madden K, Swaleh R, Goslings JC, Petrisor B, et al. Barriers to and facilitators for screening women for intimate partner violence in surgical fracture clinics: a qualitative descriptive approach. BMC Musculoskelet Disord. 2013;14:122.
22. Spedding RL, McWilliams M, McNicholl BP, Dearden CH. Markers for domestic violence in women. J Accid Emerg Med. 1999;16(6):400-2.
23. Furbee PM, Sikora R, Williams JM, Derk SJ. Comparison of domestic violence screening methods: a pilot study. Ann Emerg Med. 1998;31(4):495-501.
24. Curnow SA. The Open Window Phase: helpseeking and reality behaviors by battered women. Appl Nurs Res. 1997;10(3):128-35.
25. Gardezi M, Moore HG, Rubin LE, Grauer JN. Predictors of Physical Abuse in Elder Patients With Fracture. J Am Acad Orthop Surg Glob Res Rev. 2022;6(7).
26. Sprague S, Madden K, Dosanjh S, Godin K, Goslings JC, Schemitsch EH, et al. Intimate partner violence and musculoskeletal injury: bridging the knowledge gap in orthopaedic fracture clinics. BMC Musculoskelet Disord. 2013;14:23.

Figure Legends

Figure 1. The organisation of data collection Study packages were assembled upon triage (1) and patients were asked to fill out the forms A and B in private in the examination room and put them in a sealed envelope

For peer review only - http://bmjopen.bmj.com/site/about/guidelines.xhtml

DORIS

(2). Forms containing questions and study information were re-collected and stored in the ED (3A) in order to diminish spread of word about the study. Sealed envelopes containing forms A and B were collected daily by the study research nurse (3B). The research nurse identified patients who wished to meet a welfare officer and booked them to the trauma clinic before data was inputted by the research group (4).
Abbreviations: ED = emergency department

Figure 2. Flow chart of study inclusion

Figure 3. Occurrence and type of abuse among patients reporting on DV in a current relationship
Note: Three of the 95 patients reporting on DV were not in a current relationship and an additional three patients did not fill in the questions about abuse in their relationship.

For peer review only

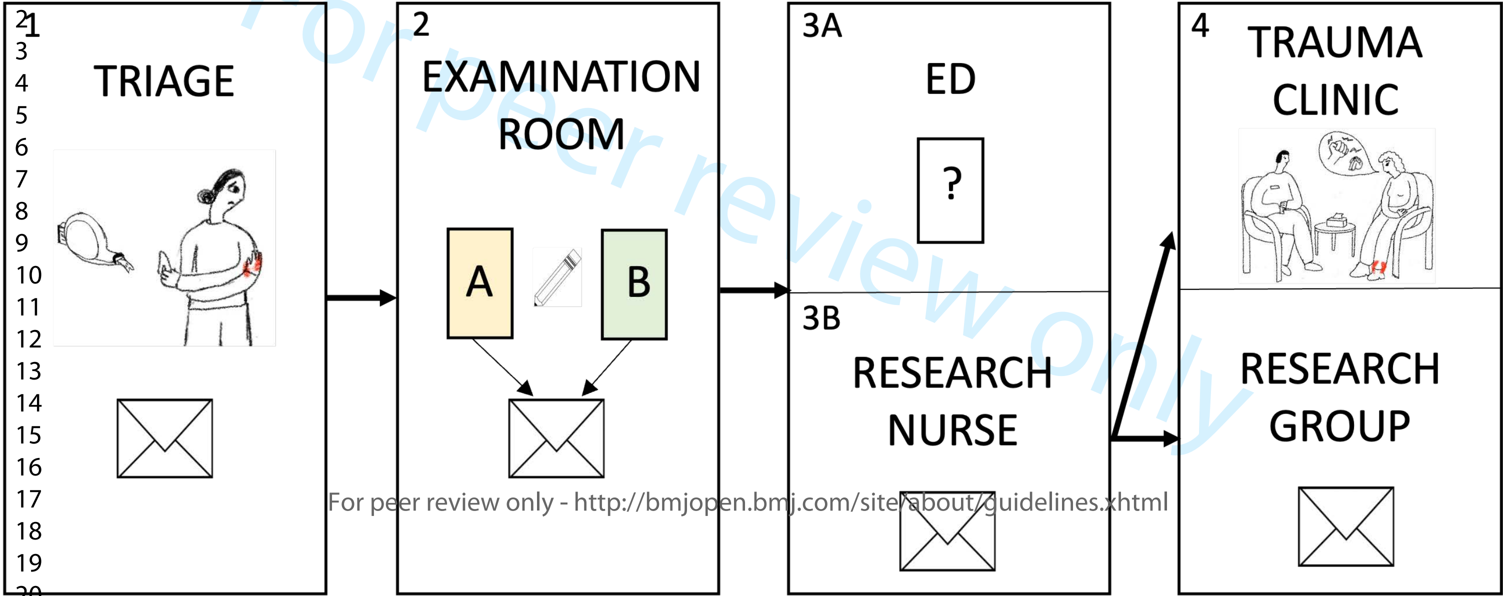

For peer review only - http://bmjopen.bmj.com/site/about/guidelines.xhtml

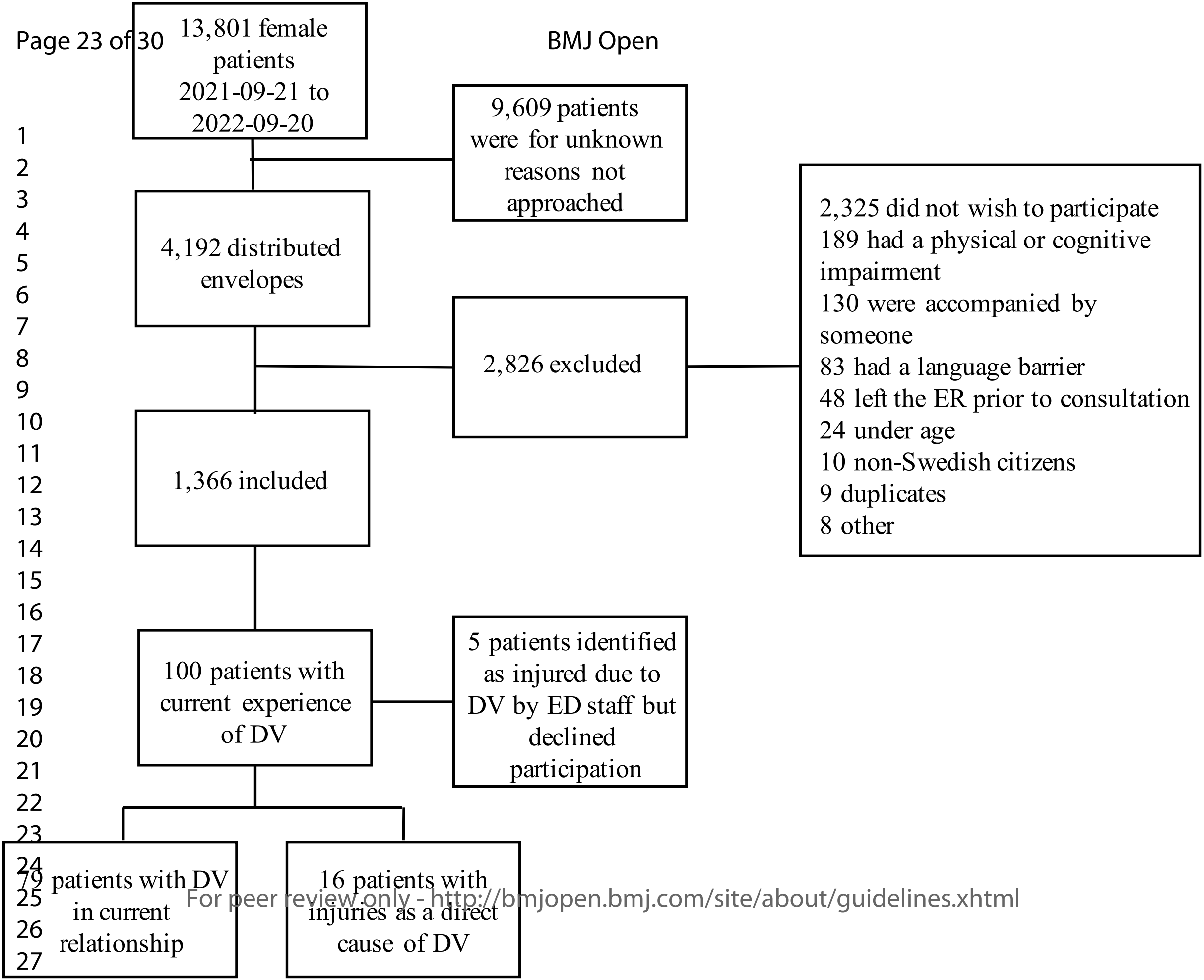

For peer review only - http://bmjopen.bmj.com/site/about/guidelines.xhtml

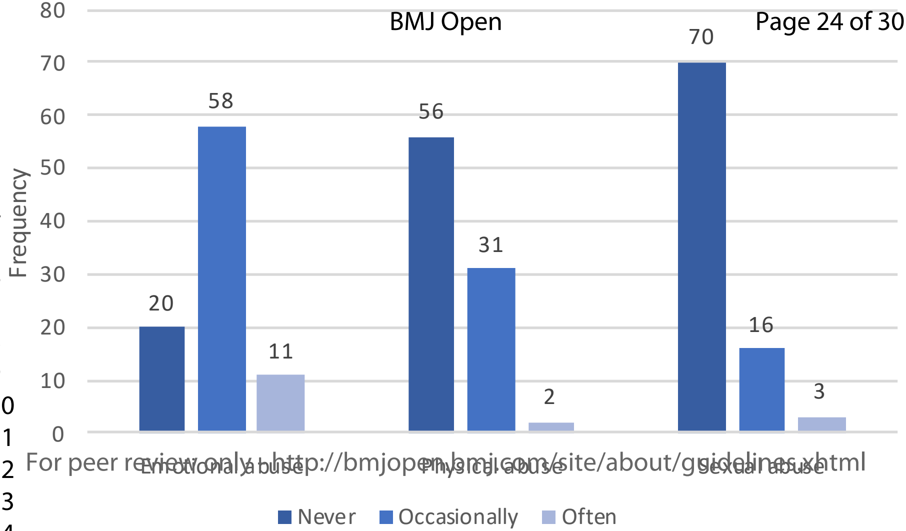
Frequency
80
70
60
50
40
30
20
10
0
20
58
11
56
31
2
70
16
3
Emotional abuse
Physical abuse
Sexual abuse
Never
Occasionally
Often

DORIS

In order to complete the study in the most optimal way, we would be grateful if you would do your best to answer all the questions.

What we mean by violence in close relationships is the emotional, physical or sexual violence that someone you feel emotionally close to (in the household, family or partner relationship) has subjected you to.

Answer using answer sheet A (yellow):

1. **How long have you been together with your current partner?**
(0) Less than 1 year (1) 1-5 years (2) 6-10 years (3) More than 10 years (4) I have no partner

If you have answered ***that you do not have a partner*** *you can skip to question 7*

2. **Is your current partner biologically:**
(0) Man (1) Woman (2) I prefer not to answer

3. **Has your current partner ever subjected you to physical violence?**
Physical violence includes, for example, pushing, hitting, scratching.
(0) Never (1) Occasionally (2) Often

4. **Has your current partner ever subjected you to emotional violence?**
Emotional violence includes, for example, threats, insults, controlling of social contacts.
(0) Never (1) Occasionally (2) Often

5. **Has your current partner ever subjected you to sexual violence?**
Sexual violence includes, for example, sexual humiliation, assault, rape.
(0) Never (1) Occasionally (2) Often

6. **Are you currently seeking medical care for an injury caused by your *current* partner?**
(0) Yes (1) No (2) I prefer not to answer

7. **Are you currently seeking medical care for an injury caused by violence from another person?**
(0) Yes (1) No (2) I prefer not to answer

8. **If you answered yes, what is your relationship with the person who committed the violence?**

9. **Was the person who committed the violence under the influence of any substance when they hurt you?**
(0) Yes, alcohol (1) Yes, drugs (2) I do not know (3) No

10. **Have you previously been seeking medical care for an injury caused by violence in a close relationship?**
(0) Yes: (*circle this if it happened in the current/past relationship*) (1) No (2) I prefer not to answer

11. **Do you think it is important that health care services ask about violence in close relationships?**
(0) Yes (1) No

12. **What is your level of education?**
(0) Primary school (1) High School (2) College/University

13. If you have been subjected to violence in a **current** close relationship, you have the option to receive counselling with a social worker at the trauma center at Mölndal Hospital. You will be called for a return visit in 1-2 weeks and the social worker's visit will not be visible in your medical record or on the call on paper you receive at home. The call will look like a regular call for a medical appointment. If your injury also requires a medical follow-up, you will be scheduled to see the social worker after your medical appointment. The social worker is subject to existing healthcare laws. Do you wish to speak to a social worker?

For peer review only

DORIS

Answer sheet A
please check the option that best applies to you

---

1. [0] Less than 1 year [1] 1-5 years [2] 6-10 years [3] More than 10 years [4] I have no partner

*If you have answered **that you do not have a partner** you can skip to question 7!*

2. [0] Man [1] Woman [2] I prefer not to answer

3. [0] Never [1] Occasionally [2] Often

4. [0] Never [1] Occasionally [2] Often

5. [0] Never [1] Occasionally [2] Often

6. [0] Yes [1] No [2] I prefer not to answer

7. [0] Yes [1] No [2] I prefer not to answer

8. ____

9. [0] Yes, alcohol [1] Yes, drugs [2] I do not know [3] No

10. [0] Yes: currently / in the past [1] No [2] I prefer not to answer

If you have answered yes, how many times have you been seeking medical care: ___________

11. [0] Yes [1] No

12. [0] Primary school [1] High School [2] College/University

13. [0] Yes [1] No

Consent to the DORIS study
I have been provided with the written information and hereby consent to the processing of my information in the DORIS study as described in the information for research participants.

Signature ______________________ Clarification of signature ______________________

National identification number ______________________ Date ______________________

For peer review only

DORIS

Questionnaire B

What we mean by violence in close relationships is the emotional, physical or sexual violence that someone you feel emotionally close to (in the household, family or partner relationship) has subjected you to.

1. Are you currently seeking medical care for an injury caused by violence in a close relationship?

YES NO

2. Do you think that health care services should routinely ask about violence in close relationships as the cause of injuries?

YES NO

For peer review only

DORIS

Answer sheet B
please circle the answer that best applies to you

---

1. YES NO

2. YES NO

For peer review only

DORIS

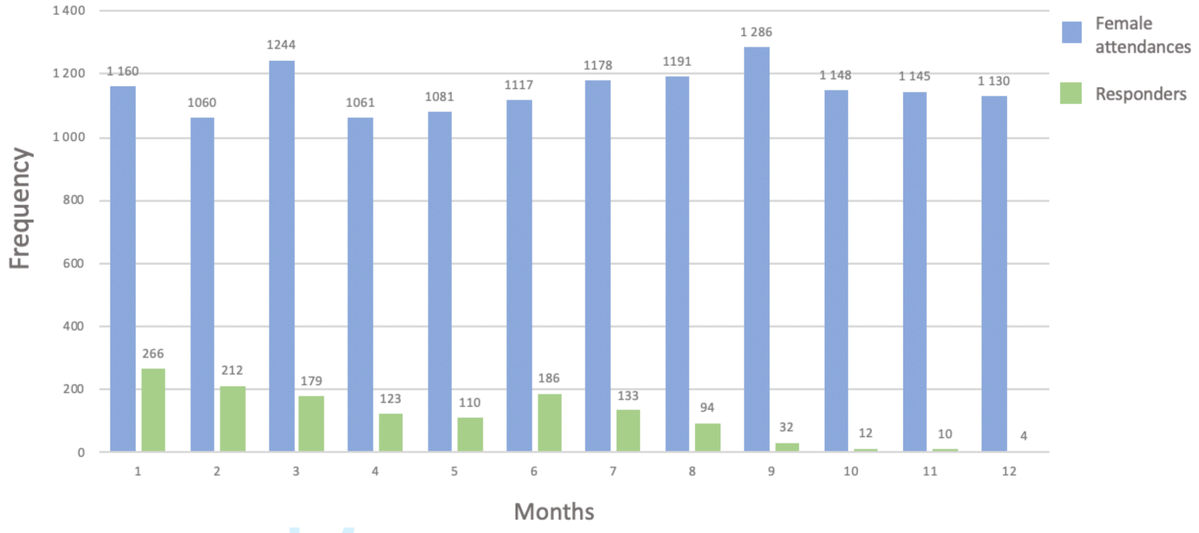

Supplementary material, Figure S1. The responder frequency per month The number of unique female attendances (blue) and responders (green) per study month (21st to 21st of the next calendar month).

For peer review only

STROBE Statement—Checklist of items that should be included in reports of ***cohort studies***

	Item No	Recommendation	Page No
**Title and abstract**	1	(*a*) Indicate the study's design with a commonly used term in the title or the abstract<br>(*b*) Provide in the abstract an informative and balanced summary of what was done and what was found	1
**Introduction**			
Background/rationale	2	Explain the scientific background and rationale for the investigation being reported	4
Objectives	3	State specific objectives, including any prespecified hypotheses	4, 6
**Methods**			
Study design	4	Present key elements of study design early in the paper	5
Setting	5	Describe the setting, locations, and relevant dates, including periods of recruitment, exposure, follow-up, and data collection	5
Participants	6	(*a*) Give the eligibility criteria, and the sources and methods of selection of participants. Describe methods of follow-up<br>(*b*) For matched studies, give matching criteria and number of exposed and unexposed	6
Variables	7	Clearly define all outcomes, exposures, predictors, potential confounders, and effect modifiers. Give diagnostic criteria, if applicable	6
Data sources/ measurement	8*	For each variable of interest, give sources of data and details of methods of assessment (measurement). Describe comparability of assessment methods if there is more than one group	6
Bias	9	Describe any efforts to address potential sources of bias	6
Study size	10	Explain how the study size was arrived at	-
Quantitative variables	11	Explain how quantitative variables were handled in the analyses. If applicable, describe which groupings were chosen and why	6
Statistical methods	12	(*a*) Describe all statistical methods, including those used to control for confounding<br>(*b*) Describe any methods used to examine subgroups and interactions<br>(*c*) Explain how missing data were addressed<br>(*d*) If applicable, explain how loss to follow-up was addressed<br>(*e*) Describe any sensitivity analyses	6
**Results**			
Participants	13*	(a) Report numbers of individuals at each stage of study—eg numbers potentially eligible, examined for eligibility, confirmed eligible, included in the study, completing follow-up, and analysed<br>(b) Give reasons for non-participation at each stage<br>(c) Consider use of a flow diagram	7
Descriptive data	14*	(a) Give characteristics of study participants (eg demographic, clinical, social) and information on exposures and potential confounders<br>(b) Indicate number of participants with missing data for each variable of interest<br>(c) Summarise follow-up time (eg, average and total amount)	7
Outcome data	15*	Report numbers of outcome events or summary measures over time	7

For peer review only

For peer review only - http://bmjopen.bmj.com/site/about/guidelines.xhtml

Main results	16	(*a*) Give unadjusted estimates and, if applicable, confounder-adjusted estimates and their precision (eg, 95% confidence interval). Make clear which confounders were adjusted for and why they were included<br>(*b*) Report category boundaries when continuous variables were categorized<br>(*c*) If relevant, consider translating estimates of relative risk into absolute risk for a meaningful time period	-
Other analyses	17	Report other analyses done—eg analyses of subgroups and interactions, and sensitivity analyses	-
**Discussion**			
Key results	18	Summarise key results with reference to study objectives	9
Limitations	19	Discuss limitations of the study, taking into account sources of potential bias or imprecision. Discuss both direction and magnitude of any potential bias	9-12
Interpretation	20	Give a cautious overall interpretation of results considering objectives, limitations, multiplicity of analyses, results from similar studies, and other relevant evidence	13
Generalisability	21	Discuss the generalisability (external validity) of the study results	13
**Other information**			
Funding	22	Give the source of funding and the role of the funders for the present study and, if applicable, for the original study on which the present article is based	14

*Give information separately for exposed and unexposed groups.

Note: An Explanation and Elaboration article discusses each checklist item and gives methodological background and published examples of transparent reporting. The STROBE checklist is best used in conjunction with this article (freely available on the Web sites of PLoS Medicine at http://www.plosmedicine.org/, Annals of Internal Medicine at http://www.annals.org/, and Epidemiology at http://www.epidem.com/). Information on the STROBE Initiative is available at http://www.strobe-statement.org.

For peer review only

For peer review only - http://bmjopen.bmj.com/site/about/guidelines.xhtml

BMJ Open

The DORIS study: Domestic violence in ORthopaedIcS, a prospective cohort study at a Swedish hospital on the annual prevalence of domestic violence in orthopaedic emergency care

Journal:	*BMJ Open*
Manuscript ID	bmjopen-2024-085618.R2
Article Type:	Original research
Date Submitted by the Author:	26-Apr-2024

Complete List of Authors:	Svensson Malchau, Karin; Sahlgrenska University Hospital, Orthopaedics<br>Caragounis, Eva-Corina; University of Gothenburg, Department of Surgery, Institute of Clinical Sciences<br>Sundfeldt, Mikael; Sahlgrenska University Hospital, Orthopaedics

**Primary Subject Heading**:	Epidemiology
Secondary Subject Heading:	Public health, Emergency medicine, Patient-centred medicine
Keywords:	ACCIDENT & EMERGENCY MEDICINE, Awareness, Health, Mass Screening, ORTHOPAEDIC & TRAUMA SURGERY

SCHOLARONE™
Manuscripts

I, the Submitting Author has the right to grant and does grant on behalf of all authors of the Work (as defined in the below author licence), an exclusive licence and/or a non-exclusive licence for contributions from authors who are: i) UK Crown employees; ii) where BMJ has agreed a CC-BY licence shall apply, and/or iii) in accordance with the terms applicable for US Federal Government officers or employees acting as part of their official duties; on a worldwide, perpetual, irrevocable, royalty-free basis to BMJ Publishing Group Ltd ("BMJ") its licensees and where the relevant Journal is co-owned by BMJ to the co-owners of the Journal, to publish the Work in this journal and any other BMJ products and to exploit all rights, as set out in our licence.

The Submitting Author accepts and understands that any supply made under these terms is made by BMJ to the Submitting Author unless you are acting as an employee on behalf of your employer or a postgraduate student of an affiliated institution which is paying any applicable article publishing charge ("APC") for Open Access articles. Where the Submitting Author wishes to make the Work available on an Open Access basis (and intends to pay the relevant APC), the terms of reuse of such Open Access shall be governed by a Creative Commons licence – details of these licences and which Creative Commons licence will apply to this Work are set out in our licence referred to above.

Other than as permitted in any relevant BMJ Author's Self Archiving Policies, I confirm this Work has not been accepted for publication elsewhere, is not being considered for publication elsewhere and does not duplicate material already published. I confirm all authors consent to publication of this Work and authorise the granting of this licence.

For peer review only

DORIS

The DORIS study: Domestic violence in ORthopaedIcS, a prospective cohort study at a Swedish hospital on the annual prevalence of domestic violence in orthopaedic emergency care

Karin Svensson Malchau MD PhD[1], Eva-Corina Caragounis MD PhD[2], Mikael Sundfeldt MD PhD[1]

[1]Department of Orthopaedics, Institute of Clinical Sciences, Sahlgrenska University Hospital, Sahlgrenska Academy, University of Gothenburg, Gothenburg, Sweden
[2]Department of Surgery, Institute of Clinical Sciences, Sahlgrenska University Hospital, Sahlgrenska Academy, University of Gothenburg, Gothenburg, Sweden

Corresponding author: Karin Svensson Malchau, Department of Orthopaedics, R-huset, Länsmansvägen 28, 431 42 Mölndal, Sweden. E-mail address: karin.am.svensson@vgregion.se, telephone number +46(0)313430761.

For peer review only

For peer review only - http://bmjopen.bmj.com/site/about/guidelines.xhtml

DORIS

ABSTRACT

Background

Domestic violence (DV) is a major problem which despite many efforts persists globally. Victims of DV can present with various injuries, whereof musculoskeletal presentation is common.

Objectives

The DORIS study aimed to establish the annual prevalence of DV at an orthopaedic emergency department (ED) in Sweden.

Design

Female adult patients with orthopaedic injuries seeking treatment at a tertiary orthopaedic centre between September 2021–2022 were screened during their ED visit.

Setting

A single-centre study at a tertiary hospital.

Participants

Adult female patients seeking care for acute orthopaedic injuries were eligible for the study. During the study period, 4,192 female patients were provided with study forms and 1,366 responded (32.5%).

Primary and secondary outcome measures

The primary outcome measure was to establish the annual prevalence of injuries due to DV and secondly, establish the rate of current experience of any type of DV.

Results

One in 14 had experience of current DV (n=100, 7.5%) and one in 65 (n=21, 1.5%) had an injury due to DV.

For peer review only - http://bmjopen.bmj.com/site/about/guidelines.xhtml

DORIS

Conclusions

The prevalence of DV found in the current study is comparable to international findings and adds to the growing body of evidence that it needs to be considered in clinical practice. It is important to raise awareness of DV, and frame strategies, as healthcare staff have a unique position to identify and offer intervention to DV victims.

ARTICLE SUMMARY

Strengths and limitations of this study

- This is a prospective observational study investigating the annual prevalence of domestic violence (DV) in female orthopaedic patients using questionnaires containing validated questions for DV.
- Study participants were approached individually without the presence of company and great discretion was taken to ascertain the safety of DV victims.
- The study was designed to screen all female patients consecutively, and although difficulties in the practical implementation of the screening program impeded the desired inclusion rate, a large volume of patients were included.
- Study participants could not choose to be anonymous which may have deterred some patients from filling out the study questionnaires.

Keywords: orthopaedics, domestic violence, trauma

Introduction

Domestic violence (DV) is a serious public health problem estimated to affect as many as 27% of women in partner relationships during their lifetime.[1] It is an insidious process, starting off with phases of systematic psychological abuse often leading to physical abuse.[2] Aside its societal and individual economic consequences,[3] it is one of the most common

For peer review only - http://bmjopen.bmj.com/site/about/guidelines.xhtml

causes for physical injuries in women and victims are at great risk for mental health issues, suicide and homicide.[4] 20–50% of female homicides, are caused by a former or current intimate partner,[5, 6] and in Sweden, the death toll due to known DV was 13 in 2020.[7]

Musculoskeletal injuries are one of the most common presentations of DV.[8, 9] One in 50 women present to fracture clinics with an injury due to DV.[10] Recognition of DV as an injury mechanism is important and orthopaedic units have been suggested ideal for screening.[11, 12] However, the difficulties of identifying DV are many. Victims may be prevented from seeking medical attention by their abuser which was found true for 36% of women in Canada.[8] A further challenge is the absence of active questioning in healthcare and that patients may not disclose occurrence of abuse.[13] Orthopaedic surgeons under-estimate the prevalence of DV,[14] and do not ask about DV.[10]

Implementation of screening within healthcare may lead to a greater detection of DV, which in turn can be potentially lifesaving. Nevertheless, questioning for DV is not standard and formal documentation is poor.[15] Sweden is considered the most gender equal country in the European Union,[7] however, research on DV in orthopaedics is scarce and little is known about its prevalence in Sweden. The current project aimed to identify the annual prevalence of orthopaedic injuries caused by DV and current experience of DV, in female patients at the largest orthopaedic emergency department (ED) in Sweden. Types of DV, injury due to DV and stated injury mechanisms were also evaluated.

Methods

Study design

For peer review only

For peer review only - http://bmjopen.bmj.com/site/about/guidelines.xhtml

DORIS

This is a self-reported questionnaire-based study including questions validated for detection of partner violence in an orthopaedic setting.[11]

Objectives

The primary objective was to identify the annual prevalence of orthopaedic injuries sustained directly due to DV. The secondary objectives were to establish the annual prevalence of current experience of DV and investigate which types of DV, injuries and stated injury mechanisms were most common.

Setting

The study was conducted at the ED of the Sahlgrenska University Hospital/Mölndal in Gothenburg, Sweden from 21st September 2021 to 21st September 2022. The ED averages 45,000 unique attendances yearly and the orthopaedic section has an average of 38 female attendances daily.

Sets of study information, marked with name and social security number, were assembled upon triage. Staff were instructed to hand out the forms to all female patients fulfilling the study inclusion criteria. Forms were handed out in the examination room, filled out in private, and put in a sealed envelope (Figure 1). ED staff were unaware of status of study participation. The forms were contained inside the ED as a precautious measure to diminish the risk of unauthorized persons identifying potential victims. If ED staff discovered a case of DV when informing patients about the study, they were asked to mark the envelope with an "X". However, the patient was only included in the further analysis if she consented to study participation. Medical records of consenting patients reporting DV were reviewed to assess injury type and severity.

For peer review only - http://bmjopen.bmj.com/site/about/guidelines.xhtml

Patients who wished to meet a project counsellor were booked for a medical follow-up without mention of the counsellor. This was intentional to protect the patient in cases of cohabitation with the abuser.

Participants

Patients of female sex of at least 18 years of age and with residency in Sweden triaged to the orthopaedic section of the ED were included in the study. Patients accompanied by someone, or with cognitive impairment or physical impairment, i.e. dementia or poor eyesight, were excluded. Furthermore, patients who could not understand Swedish, English or Arabic were also excluded. No sample size calculation was conducted as the objective was to establish the annual prevalence of DV victims.

Study questionnaire

Screening was performed using paper questionnaires, which had been developed based on the work of Sprague et al., where the direct questioning approach detected DV to a greater extent than other tools evaluated for orthopaedic use .[11] Additional questions on demography were added (see Supplementary material). There were two forms (A and B) of which B was simplified and more anonymous in order to encourage higher responder rates (Supplementary material, S1). Participants received both forms and could choose which form to fill in. Study forms were provided in Swedish and translated two-way in English and Arabic.

Definitions

For peer review only

DV was defined as emotional, physical or sexual abuse. Any occurrence within the family, domestic unit or by former intimate partners, was included, as defined by the Istanbul Convention (2011).[16] A relationship was defined as a partnership lasting at least one month.

	**All responders** (n=1,361)	**Responders reporting no experience of DV in current relationship** (n=1,165)	**Responders reporting current DV, not DV as direct cause of injury** (n=79)	**Responders reporting DV as direct cause of injury** (n=16)	**Responders with missing or incomplete answers on current DV** (n=101)

Data analysis

Data was analysed descriptively with frequency counts and percentages for categorical variables. Software IBM SPSS version 29 was used for data analysis.

Ethical considerations

Written consent was obtained upon study enrolment in line with the Ethical Review Board's regulations (DNR 2021-01752).

Patient and Public Involvement

It was not deemed appropriate to involve patients or the public in the design, or conduct, or reporting, or dissemination plans of our research.

Results

In total, 4,192 (30.4%) out of 13,801 unique female attendances registered at the orthopaedic section of the ED were given study forms. Of these, 1,366 (32.6%) agreed to inclusion (Figure 2). The majority of responders spoke Swedish (99.4%), did not live in a socially disadvantaged area (80.4%) and were in a relationship (62.2%) (Table 1).

For peer review only

DORIS

**Age** (range, years)					
18-29	226 (16.6)	201 (17.3)	8 (10.1)	2 (12.5)	15 (14.8)
30-39	211 (15.5)	182 (15.6)	13 (16.5)	4 (25.0)	12 (11.9)
40-49	211 (15.5)	174 (14.9)	17 (21.5)	4 (25.0)	16 (15.8)
50-59	262 (19.3)	234 (20.1)	15 (19.0)	1 (6.3)	12 (11.9)
60-69	239 (17.6)	199 (17.1)	17 (21.5)	4 (25.0)	19 (18.8)
>70	202 (14.8)	167 (14.3)	8 (10.1)	1 (6.3)	26 (25.7)
Missing	10 (0.7)	8 (0.7)	1 (1.3)	0 (0.0)	1 (1.0)
**Resident of a socially disadvantaged area**					
Yes	227 (16.7)	184 (15.8)	16 (20.3)	3 (18.8)	24 (23.8)
No	1101 (80.9)	954 (81.9)	58 (73.4)	13 (81.3)	75 (75.2)
Protected person/not a resident in Gothenburg	9 (0.7)	6 (0.1)	3 (3.8)	0 (0.0)	0 (0.0)
Missing	24 (1.8)	21 (1.8)	2 (2.5)	0 (0.0)	1 (1.0)
**Language**					
Swedish	1353 (99.4)	1161 (99.7)	77 (97.5)	15 (94.1)	100 (99.0)
English	5 (0.4)	3 (0.3)	1 (1.3)	0 (0.0)	1 (1.0)
Arabic	3 (0.2)	1 (0.0)	1 (1.3)	1 (5.9)	0 (0.0)
**Education level**					
Compulsory school	104 (7.6)	82 (7.0)	5 (6.3)	2 (12.5)	15 (14.9)
High school	459 (33.7)	395 (33.9)	30 (38.0)	9 (56.3)	25 (24.8)
University	727 (53.4)	650 (55.8)	43 (54.4)	4 (25.0)	29 (28.7)
Missing	71 (5.3)	38 (3.3)	1 (1.3)	1 (6.3)	31 (30.7)
**Partner sex**					
No partner	430 (31.6)	427 (36.7)	0 (0.0)	3 (17.6)	0 (0.0)
Male	806 (59.2)	711 (61.0)	75 (95.0)	10 (64.7)	10 (10.0)
Female	40 (2.9)	22 (2.0)	2 (2.5)	2 (11.8)	14 (13.9)
Missing	85 (6.2)	5 (0.4)	2 (2.5)	1 (5.9)	77 (76.2)
**Duration of relationship**					
Less than one year	36 (2.6)	30 (2.6)	4 (5.1)	2 (12.5)	1 (1.0)
1-5years	165 (12.1)	144 (12.4)	12 (15.2)	3 (18.8)	6 (5.9)
6-10 years	96 (7.1)	80 (6.9)	12 (15.2)	2 (12.5)	2 (2.0)
More than 10 years	567 (41.7)	476 (40.9)	50 (63.3)	5 (31.3)	36 (35.6)
No partner	440 (32.3)	430 (36.9)	1 (1.3)	3 (18.8)	6 (5.9
Missing	57 (4.2)	5 (0.4)	0 (0.0)	2 (12.5)	50 (50.0)
**Have you ever sought medical care for DV?**					
No	1050 (77.1)	942 (80.9)	57 (72.2)	8 (50.0)	43 (42.6)
Yes	54 (4.0)	38 (3.3)	10 (12.7)	3 (18.8)	4 (4.0)
Missing	257 (18.9)	185 (15.9)	13 (16.5)	5 (31.3)	54 (53.5)
**Should health care workers ask about DV?**					
Yes	1209 (88.8)	1068 (91.7)	76 (96.2)	14 (87.5)	51 (50.5)
No	41 (3.0)	30 (2.6)	2 (2.5)	0 (0.0)	9 (8.9)
Missing	111 (8.2)	67 (5.8)	1 (1.3)	2 (12.5)	41 (40.6)

Table 1. Demographics of all responders and whether health care should ask about DV grouped by experience of DV.

Experience of DV

For peer review only

DORIS

Of the 1,366 patients, 100 patients (7.5%) had current experience of DV and 21 (1.5%) of them had an injury due to DV. Of the 21 patients, 16 consented to filling out the study forms. The remaining five patients disclosed DV to healthcare staff but declined to fill out the study forms. Therefore, they were not included in the further analysis, leaving 95 patients of the 100 patients who had stated current experience of DV, eligible for further analysis (Figure 2).

	Frequency n (%)
**Formal documentation of DV in medical record**	
Yes	8 (50)
No	8 (50
**Stated injury mechanism in medical record**	
Fall trauma, unspecified	8 (50)
Abuse	8 (50)
**Orthopaedic treatment**	
Pain medication and physiotherapy	8 (50)

DV (any type) was reported by 89 (89/1361, 6.5%) patients in their current relationship. Emotional abuse was most common (69/89, 77.5%) followed by physical abuse (33/89, 37.1%) and sexual abuse (19/89, 21.3%) (Figure 3).

DV as a direct cause of injury

In total, 21 patients with an injury due to DV were identified (Figure 2), meaning that one in 65 patients needed medical attention due to physical abuse. Of the 16 consenting DV victims, eight had previously been in contact with healthcare for an injury due to abuse. Formal documentation of DV was noted in eight medical records, and in the remaining cases the injury mechanism was unspecified fall trauma (Table 2).

For peer review only

For peer review only - http://bmjopen.bmj.com/site/about/guidelines.xhtml

Table 2. Injury mechanism as stated in the medical records and treatment needs due to DV

Immobilization (cast/orthosis)	6 (38)
Surgery	2 (12)
**Need for sick leave**	
Yes	5 (31)
No	11 (69)

The age span of DV victims was 18–76 years. Three patients were from socially disadvantaged areas and three patients had female partners. The majority of patients had completed high school but had no further academic education (Table 1). Eight patients reported on repeated abuse in their current relationship of which five stated an occurrence of both emotional, physical, and sexual abuse.

Fractures were the most prevalent injury followed by contusions and joint distortions (Table 3). Five patients sustained injuries requiring sick leave and two patients required surgery (Table 3). Thirty-seven follow-up visits were recorded due to DV injuries (excluding visits to the counsellor).

For peer review only

DORIS

Table 3. Type of injuries noted in cases with DV as direct cause of injury

**Injury type and localisation**	**Frequency** n (%)
**Fracture**	6 (38)
Hand	5
Foot	1
**Contusion**	4 (25)
Upper extremity	1
Lower extremity	3
**Distortion**	4 (25)
Shoulder	1
Knee	1
Foot	2
**Joint dislocation**	1 (6)
**Ligament rupture**	1 (6)
**Laterality of injury**	
Right	10 (63)
Left	4 (25)
Missing	2 (12)

For peer review only

Screening for DV

In total, 1,208 women (89.0%) were of the opinion that healthcare staff should ask about DV (Table 1). However, two of the 16 patients (12.5%) injured due to DV did not feel that screening was necessary. Fifty-four patients (4.0%) had previously contacted healthcare for physical abuse (Table 1), whereof 34 of these patients were still in an abusive relationship.

DORIS

The project counsellors had contact with 23 patients whereof 8 had been injured due to DV. 12 patients (52.2%) showed up for their appointment. Six patients failed to appear, four had misunderstood or were too injured to come for their appointment. One patient had given a faulty address and did not respond to phone calls.

Discussion

The DORIS study aimed to establish the prevalence of injuries directly caused by DV, current experience of DV, types of DV, injuries and stated injury mechanisms in female patients in the largest orthopedic ED in Northern Europe. It also evaluated the rate of types of DV, injuries due to DV and what injury mechanisms were stated by victims. A rate of one in 14 patients (100/1366, 7.5%) with current experience of DV and one in 65 patients (21/1366, 1.6%) injured due to DV was established.

The prevalence of injuries due to DV (1.6%) is within the span of prevalence reported by the PRAISE group (0-3%), who conducted a multi-national investigation of intimate partner violence in female patients at orthopaedic injury clinics.[10] Current experience of DV was recorded in the DORIS study whereas previous studies have investigated the 12-month prevalence. A 12-month prevalence of DV of 15–22% in orthopaedic patients has previously been reported.[10, 17] In the DORIS study, 6.5% (89/1366) experienced DV in a current partner relationship. Differences in recruitment methods, study settings and staff engagement could serve as explanations to the lower prevalence in Sweden. The lower prevalence may also reflect governmental and societal policies on gender equality in Sweden.

For peer review only - http://bmjopen.bmj.com/site/about/guidelines.xhtml

DORIS

When comparing proportions of type of abuse, the present study established that emotional abuse was most common. This is also true in Scottish, American and Canadian settings.[10, 17] However, surprisingly, in the Netherlands and in Denmark, countries seemingly more comparable to Sweden, physical abuse was most common.[10] It may be difficult to understand what is meant by emotional abuse, the DORIS study forms contained examples of emotional abuse which may explain the higher prevalence.

Formal documentation of DV was noted in 50% of cases, meaning that 50% were not identified in the regular healthcare setting. Routine screening of DV leads to higher detection rates,[18] however, only 2% of healthcare workers in orthopedics routinely ask about it.[19] Surgeons feel uncomfortable and unsure of what to do if their patient is a victim which calls for better education and support models within healthcare.[20, 21]

Although it is important to be suspicious of inconsistent injury mechanisms or “red flags”, such as falling down the stairs,[22] feasible injury mechanisms were disclosed in 50% of the DV cases. Hence, questioning for DV should not just be conducted when suspicion is raised, as is often the case. Within the DORIS study, direct questioning, in questionnaire format, was used as this has proven efficient for DV screening and is less time consuming in an ED setting.[11, 23] However, the study forms contained a lot of text due to regulations stated by the Ethical Review Board, which may have discouraged potential responders. In the continued work of improving DV detection at the study site efforts will be made to optimise the screening tool.

Merely 50% of patients with an injury due to DV had previously been in contact with healthcare for DV. Hence, the remaining patients may have presented with an index injury. This finding supports the, previously suggested,[11, 19] need for screening in orthopaedic

For peer review only

DORIS

settings, as early intervention can be potentially lifesaving. Up to 81% of female patients are of the opinion that healthcare staff should ask about DV.[10, 19, 23] The corresponding numbers were somewhat higher in the DORIS study (89% in the entire cohort and 94-96% in abused patients). Cultural differences and thereby expectations on healthcare may explain the aforementioned variances.

The strength of the DORIS study is its setting at the largest orthopaedic ED in Northern Europe. After, the PRAISE study,[10] DORIS is the largest prevalence study in orthopedics. Due to COVID restrictions during the study period, company was generally not allowed in the ED which facilitated the distribution of study forms. Victims of DV were also offered follow-up with a counsellor within the study.

A major limitation may be nonresponse bias. Although the study was regarded important by ED staff, the distribution rate of study forms was 30% and response rate 33%. The authors had meetings with ED staff and two counsellors were recruited to provide an in-house support program to increase the likelihood for staff engagement.[19] Unfortunately, due to management issues, the staffing situation became more turbulent with several experienced nurses and assistant nurses choosing to resign throughout the year. The authors believe that the inconsistencies in staffing were the main reason for poor study enrolment (Supplementary material, Figure 1). In addition, despite being an excellent forum for DV screening,[18] in regard to the "open window phase" (in which victims may be more receptive and prone to seek help after abuse),[24] the ED as such is a busy and stressful place. In general, detecting DV may be difficult in such a setting: staff may be unaware of DV as a problem, and patients may feel uncomfortable confiding in ED staff. For this reason, it is crucial to structure EDs in a

For peer review only

manner where triage can be done in private, as also suggested by Ahmad et al.,[18] and where patients are unaccompanied in triage as standard routine.

Poor response rate was partly expected. Similar studies,[10, 17] have had different approaches to recruitment making it difficult to evaluate what an acceptable response rate is. Due to the delicate nature of the study, the authors had preferred that social security number and further personal details were omitted when consenting to the study. The need to do this may have deterred potential victims from disclosing DV. However, full disclosure of personal details was a requirement from the Ethical Review Board due to research regulations. Furthermore, the authors have reason to believe that the 2,325 patients who for some reason did not wish to participate in the study may not have received proper study information or been given a chance to fill out the study forms.

The exclusion criteria imply certain limitations. Elderly patients, either accompanied by caregivers or with the diagnosis of dementia, were not included. Despite the difficulties of capturing cases in this group, it is important to acknowledge their vulnerability and that both dementia and female sex are predictive of abuse.[25] Furthermore, the authors acknowledge that DV affects both female and male patients. Screening of females was chosen as female DV patients have a greater fracture risk, 83% of ED visits due to DV are female, and 50% of female homicides are due to DV.[12] However, the long-term goal for the DORIS project is to provide a healthcare program dedicated to DV patients regardless of sex.

The DORIS study focused on current abuse, whereas previous research, such as conducted by the PRAISE group and Sardinha et al. also investigated life-time abuse.[1, 10, 17] In hindsight, the inclusion of life-time abuse would have been interesting for comparative reasons. However,

For peer review only

when designing the study, the authors decided that the patient's current situation was the most clinically relevant and therefore most important.

Despite its limitations, and a probable under-reporting of DV, the finding of one in 65 patients translates to one victim of DV injuries nearly every second day, and two to three patients with current experience of DV daily, at the study center. Interventions are essential to disrupt continued abuse and healthcare has an important role in the detection of DV.[9, 26] The experience generated by the present study suggests that screening is necessary in order to improve identification of DV cases and that patients expect healthcare to engage in detecting DV. The results from the DORIS study will be used to improve routines at the study site, and hopefully inspire to similar actions elsewhere.

Conclusion

The prevalence of DV established in the current study implies a high annual volume of DV victims at the study site. DV victims may come to an orthopaedic setting with an index injury and healthcare staff have an unique opportunity to intervene. The DORIS study adds to the growing body of evidence that DV needs attention in the healthcare setting. Increased awareness and actions to identify DV is imperative, and it is important to educate, engage and provide adequate conditions for healthcare staff to conduct screening. Future work should focus on implementing DV screening as a routine and provide a safe environment for DV victims in all healthcare disciplines.

Author contributions

For peer review only

For peer review only - http://bmjopen.bmj.com/site/about/guidelines.xhtml

KSM participated in the planning and design of the study, collection of patient data, analyzed the data, interpreted the data, drafted the manuscript, and critically revised the manuscript. ECC participated in the planning and design of the study and critically revised the manuscript. MS participated in the planning and design of the study and critically revised the manuscript.

Acknowledgements

The authors would like to thank Sandra Rosnell, Linda Stolpe, Stella Sundfeldt, Ann-Christin von Corswant, Anne Louise Gidestrand, the medical staff at the ED at Mölndal's Hospital and all the patients who chose to participate.

Funding

The study was funded by Doktor Felix Neuberghs stiftelse N/A, BGS forskningsstipendium N/A, Göteborgs Läkaresällskap N/A, SU-fonderna N/A and Konrad och Helfrid Johanssons stiftelse N/A.

Competing interest statement

All authors have completed the ICMJE uniform disclosure form at http://www.icmje.org/disclosure-of-interest/ and declare: no support from any organisation for the submitted work; no financial relationships with any organisations that might have an interest in the submitted work in the previous three years; no other relationships or activities that could appear to have influenced the submitted work

Ethical considerations

Written consent was obtained upon study enrolment in line with the Ethical Review Board's regulations (DNR 2021-01752).

For peer review only

Data availability statement

Data supporting this study cannot be shared publicly in order to protect DV victims.

References

1. Sardinha L, Maheu-Giroux M, Stockl H, Meyer SR, Garcia-Moreno C. Global, regional, and national prevalence estimates of physical or sexual, or both, intimate partner violence against women in 2018. Lancet. 2022;399(10327):803-13.
2. Murphy CM, O'Leary KD. Psychological aggression predicts physical aggression in early marriage. J Consult Clin Psychol. 1989;57(5):579-82.
3. Max W, Rice DP, Finkelstein E, Bardwell RA, Leadbetter S. The economic toll of intimate partner violence against women in the United States. Violence Vict. 2004;19(3):259-72.
4. Ellsberg M, Jansen HA, Heise L, Watts CH, Garcia-Moreno C, Health WHOM-cSoWs, et al. Intimate partner violence and women's physical and mental health in the WHO multi-country study on women's health and domestic violence: an observational study. Lancet. 2008;371(9619):1165-72.
5. Jack SPD, Petrosky E, Lyons BH, Blair JM, Ertl AM, Sheats KJ, et al. Surveillance for Violent Deaths - National Violent Death Reporting System, 27 States, 2015. MMWR Surveill Summ. 2018;67(11):1-32.
6. Stockl H, Devries K, Rotstein A, Abrahams N, Campbell J, Watts C, et al. The global prevalence of intimate partner homicide: a systematic review. Lancet. 2013;382(9895):859-65.
7. [Available from: https://eige.europa.eu/gender-equality-index/2022/domain/violence/SE.
8. Bhandari M, Dosanjh S, Tornetta P, 3rd, Matthews D, Violence Against Women Health Research C. Musculoskeletal manifestations of physical abuse after intimate partner violence. J Trauma. 2006;61(6):1473-9.
9. Giordano V, Giordano C, Lopes IM, Pires RE, Godoy-Santos A, Giannoudis PV. Orthopaedic surgeons can play important role in identifying victims of domestic violence in the emergency department - narrative review of Brazilian literature. Medicine (Baltimore). 2022;101(50):e31461.
10. Investigators P, Sprague S, Bhandari M, Della Rocca GJ, Goslings JC, Poolman RW, et al. Prevalence of abuse and intimate partner violence surgical evaluation (PRAISE) in orthopaedic fracture clinics: a multinational prevalence study. Lancet. 2013;382(9895):866-76.
11. Sprague S, Madden K, Dosanjh S, Petrisor B, Schemitsch EH, Bhandari M. Screening for intimate partner violence in orthopedic patients: a comparison of three screening tools. J Interpers Violence. 2012;27(5):881-98.
12. Loder RT, Momper L. Demographics and Fracture Patterns of Patients Presenting to US Emergency Departments for Intimate Partner Violence. J Am Acad Orthop Surg Glob Res Rev. 2020;4(2).

For peer review only

13. Brown JB, Lent B, Schmidt G, Sas G. Application of the Woman Abuse Screening Tool (WAST) and WAST-short in the family practice setting. J Fam Pract. 2000;49(10):896-903.

14. Bhandari M, Sprague S, Tornetta P, 3rd, D'Aurora V, Schemitsch E, Shearer H, et al. (Mis)perceptions about intimate partner violence in women presenting for orthopaedic care: a survey of Canadian orthopaedic surgeons. J Bone Joint Surg Am. 2008;90(7):1590-7.

15. Khurana B, Sing D, Gujrathi R, Keraliya A, Bay CP, Chen I, et al. Recognizing Isolated Ulnar Fractures as Potential Markers for Intimate Partner Violence. J Am Coll Radiol. 2021;18(8):1108-17.

16. Council of Europe Convention on preventing and combating violence against women and domestic violence. Istanbul; 2011.

17. Downie S, Kanya I, Madden K, Bhandari M, Jariwala AC. Intimate partner violence (IPV) in male and female orthopaedic trauma patients: a multicentre, cross-sectional prevalence study. BMJ Open. 2021;11(8):e046164.

18. Ahmad I, Ali PA, Rehman S, Talpur A, Dhingra K. Intimate partner violence screening in emergency department: a rapid review of the literature. J Clin Nurs. 2017;26(21-22):3271-85.

19. Downie S, Madden K, Bhandari M, Jariwala A. A prospective questionnaire-based study on staff awareness of intimate partner violence (IPV) in orthopaedic trauma patients. Surgeon. 2019;17(4):207-14.

20. Della Rocca GJ, Sprague S, Dosanjh S, Schemitsch EH, Bhandari M. Orthopaedic surgeons' knowledge and misconceptions in the identification of intimate partner violence against women. Clin Orthop Relat Res. 2013;471(4):1074-80.

21. Sprague S, Swinton M, Madden K, Swaleh R, Goslings JC, Petrisor B, et al. Barriers to and facilitators for screening women for intimate partner violence in surgical fracture clinics: a qualitative descriptive approach. BMC Musculoskelet Disord. 2013;14:122.

22. Spedding RL, McWilliams M, McNicholl BP, Dearden CH. Markers for domestic violence in women. J Accid Emerg Med. 1999;16(6):400-2.

23. Furbee PM, Sikora R, Williams JM, Derk SJ. Comparison of domestic violence screening methods: a pilot study. Ann Emerg Med. 1998;31(4):495-501.

24. Curnow SA. The Open Window Phase: helpseeking and reality behaviors by battered women. Appl Nurs Res. 1997;10(3):128-35.

25. Gardezi M, Moore HG, Rubin LE, Grauer JN. Predictors of Physical Abuse in Elder Patients With Fracture. J Am Acad Orthop Surg Glob Res Rev. 2022;6(7).

26. Sprague S, Madden K, Dosanjh S, Godin K, Goslings JC, Schemitsch EH, et al. Intimate partner violence and musculoskeletal injury: bridging the knowledge gap in orthopaedic fracture clinics. BMC Musculoskelet Disord. 2013;14:23.

For peer review only

Figure Legends

Figure 1. The organisation of data collection Study packages were assembled upon triage (1) and patients were asked to fill out the forms A and B in private in the examination room and put them in a sealed envelope

For peer review only - http://bmjopen.bmj.com/site/about/guidelines.xhtml

DORIS

(2). Forms containing questions and study information were re-collected and stored in the ED (3A) in order to diminish spread of word about the study. Sealed envelopes containing forms A and B were collected daily by the study research nurse (3B). The research nurse identified patients who wished to meet a welfare officer and booked them to the trauma clinic before data was inputted by the research group (4).
Abbreviations: ED = emergency department

Figure 2. Flow chart of study inclusion

Figure 3. Occurrence and type of abuse among patients reporting on DV in a current relationship
Note: Three of the 95 patients reporting on DV were not in a current relationship and an additional three patients did not fill in the questions about abuse in their relationship.

For peer review only

For peer review only - http://bmjopen.bmj.com/site/about/guidelines.xhtml

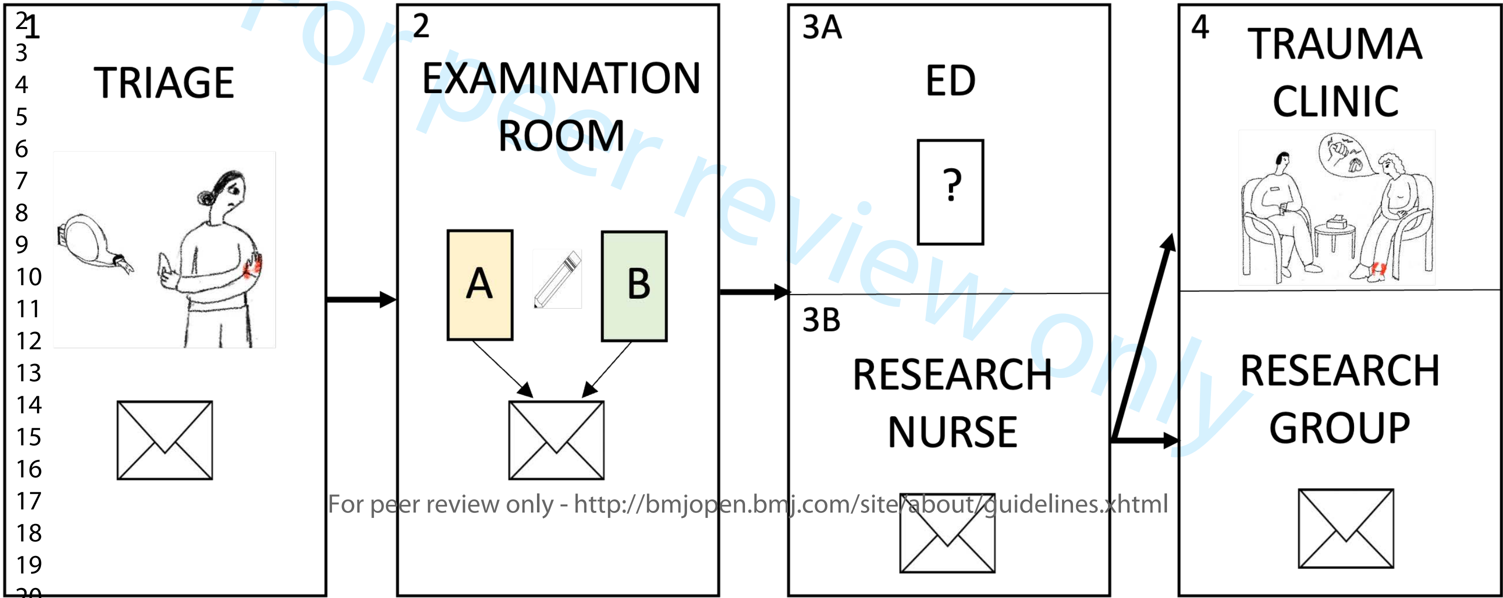

For peer review only - http://bmjopen.bmj.com/site/about/guidelines.xhtml

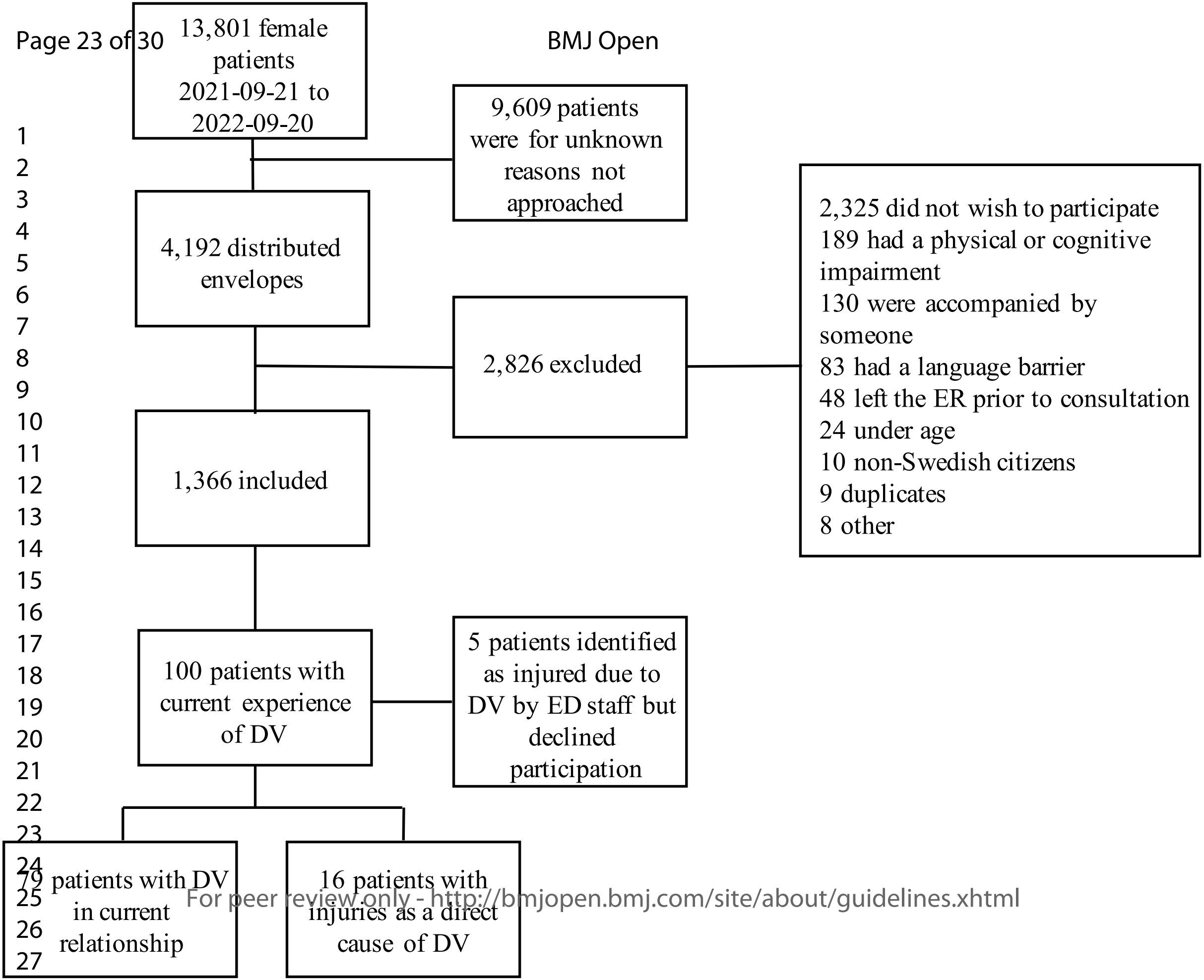

For peer review only - http://bmjopen.bmj.com/site/about/guidelines.xhtml

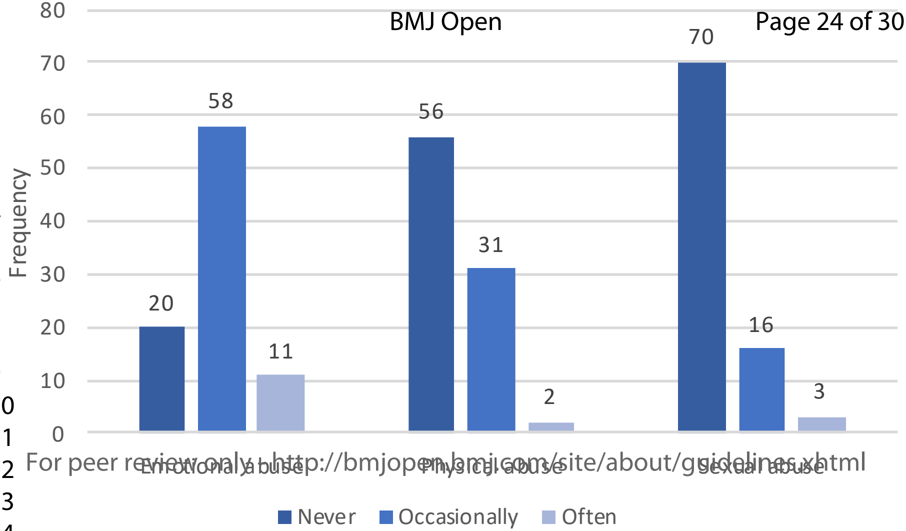
80
70
60
50
40
30
20
10
0
Frequency
20
58
11
56
31
2
70
16
3
Emotional abuse
Physical abuse
Sexual abuse
Never
Occasionally
Often

DORIS

In order to complete the study in the most optimal way, we would be grateful if you would do your best to answer all the questions.

What we mean by violence in close relationships is the emotional, physical or sexual violence that someone you feel emotionally close to (in the household, family or partner relationship) has subjected you to.

Answer using answer sheet A (yellow):

1. How long have you been together with your current partner?
(0) Less than 1 year (1) 1-5 years (2) 6-10 years (3) More than 10 years (4) I have no partner

If you have answered ***that you do not have a partner*** *you can skip to question 7*

2. Is your current partner biologically:
(0) Man (1) Woman (2) I prefer not to answer

3. Has your current partner ever subjected you to physical violence?
Physical violence includes, for example, pushing, hitting, scratching.
(0) Never (1) Occasionally (2) Often

4. Has your current partner ever subjected you to emotional violence?
Emotional violence includes, for example, threats, insults, controlling of social contacts.
(0) Never (1) Occasionally (2) Often

5. Has your current partner ever subjected you to sexual violence?
Sexual violence includes, for example, sexual humiliation, assault, rape.
(0) Never (1) Occasionally (2) Often

6. Are you currently seeking medical care for an injury caused by your *current* partner?
(0) Yes (1) No (2) I prefer not to answer

7. Are you currently seeking medical care for an injury caused by violence from another person?
(0) Yes (1) No (2) I prefer not to answer

8. If you answered yes, what is your relationship with the person who committed the violence?

9. Was the person who committed the violence under the influence of any substance when they hurt you?
(0) Yes, alcohol (1) Yes, drugs (2) I do not know (3) No

10. Have you previously been seeking medical care for an injury caused by violence in a close relationship?
(0) Yes: (*circle this if it happened in the current/past relationship*) (1) No (2) I prefer not to answer

11. Do you think it is important that health care services ask about violence in close relationships?
(0) Yes (1) No

12. What is your level of education?
(0) Primary school (1) High School (2) College/University

13. If you have been subjected to violence in a **current** close relationship, you have the option to receive counselling with a social worker at the trauma center at Mölndal Hospital. You will be called for a return visit in 1-2 weeks and the social worker's visit will not be visible in your medical record or on the call on paper you receive at home. The call will look like a regular call for a medical appointment. If your injury also requires a medical follow-up, you will be scheduled to see the social worker after your medical appointment. The social worker is subject to existing healthcare laws. Do you wish to speak to a social worker?

For peer review only

DORIS

Answer sheet A
please check the option that best applies to you

---

1. [0] Less than 1 year [1] 1-5 years [2] 6-10 years [3] More than 10 years [4] I have no partner

If you have answered ***that you do not have a partner*** *you can skip to question 7!*

2. [0] Man [1] Woman [2] I prefer not to answer

3. [0] Never [1] Occasionally [2] Often

4. [0] Never [1] Occasionally [2] Often

5. [0] Never [1] Occasionally [2] Often

6. [0] Yes [1] No [2] I prefer not to answer

7. [0] Yes [1] No [2] I prefer not to answer

8. ____

9. [0] Yes, alcohol [1] Yes, drugs [2] I do not know [3] No

10. [0] Yes: currently / in the past [1] No [2] I prefer not to answer

If you have answered yes, how many times have you been seeking medical care: ___________

11. [0] Yes [1] No

12. [0] Primary school [1] High School [2] College/University

13. [0] Yes [1] No

Consent to the DORIS study
I have been provided with the written information and hereby consent to the processing of my information in the DORIS study as described in the information for research participants.

Signature ______________________ Clarification of signature ______________________

National identification number ______________________ Date ______________________

For peer review only

DORIS

Questionnaire B

What we mean by violence in close relationships is the emotional, physical or sexual violence that someone you feel emotionally close to (in the household, family or partner relationship) has subjected you to.

1. Are you currently seeking medical care for an injury caused by violence in a close relationship?

YES NO

2. Do you think that health care services should routinely ask about violence in close relationships as the cause of injuries?

YES NO

For peer review only

DORIS

Answer sheet B
please circle the answer that best applies to you

1. YES NO

2. YES NO

For peer review only

DORIS

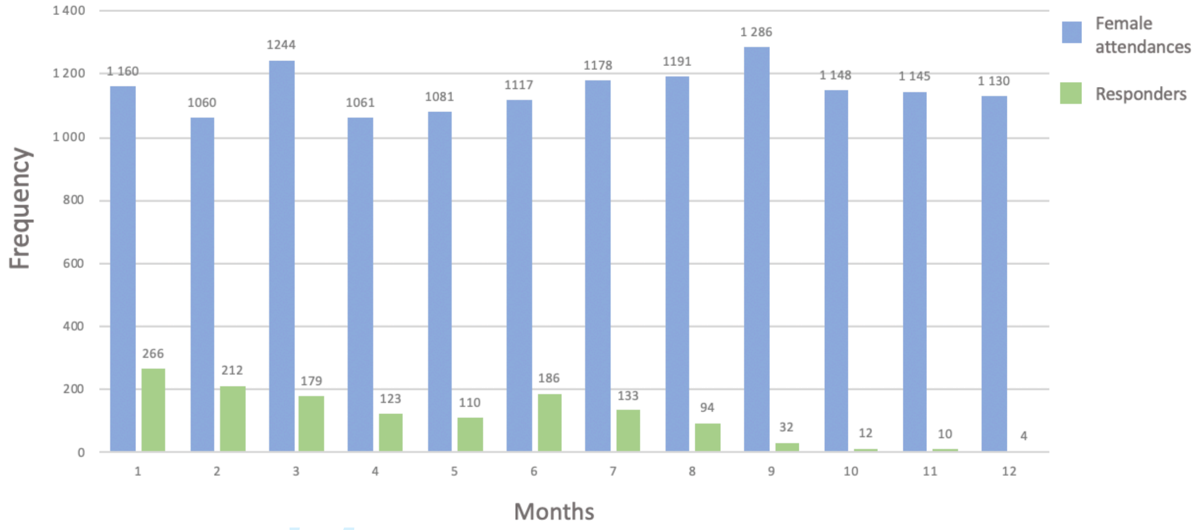

Supplementary material, Figure S1. The responder frequency per month The number of unique female attendances (blue) and responders (green) per study month (21st to 21st of the next calendar month).

For peer review only

STROBE Statement—Checklist of items that should be included in reports of ***cohort studies***

	Item No	Recommendation	Page No
**Title and abstract**	1	(*a*) Indicate the study's design with a commonly used term in the title or the abstract<br>(*b*) Provide in the abstract an informative and balanced summary of what was done and what was found	1
**Introduction**			
Background/rationale	2	Explain the scientific background and rationale for the investigation being reported	4
Objectives	3	State specific objectives, including any prespecified hypotheses	4, 6
**Methods**			
Study design	4	Present key elements of study design early in the paper	5
Setting	5	Describe the setting, locations, and relevant dates, including periods of recruitment, exposure, follow-up, and data collection	5
Participants	6	(*a*) Give the eligibility criteria, and the sources and methods of selection of participants. Describe methods of follow-up<br>(*b*) For matched studies, give matching criteria and number of exposed and unexposed	6
Variables	7	Clearly define all outcomes, exposures, predictors, potential confounders, and effect modifiers. Give diagnostic criteria, if applicable	6
Data sources/ measurement	8*	For each variable of interest, give sources of data and details of methods of assessment (measurement). Describe comparability of assessment methods if there is more than one group	6
Bias	9	Describe any efforts to address potential sources of bias	6
Study size	10	Explain how the study size was arrived at	-
Quantitative variables	11	Explain how quantitative variables were handled in the analyses. If applicable, describe which groupings were chosen and why	6
Statistical methods	12	(*a*) Describe all statistical methods, including those used to control for confounding<br>(*b*) Describe any methods used to examine subgroups and interactions<br>(*c*) Explain how missing data were addressed<br>(*d*) If applicable, explain how loss to follow-up was addressed<br>(*e*) Describe any sensitivity analyses	6
**Results**			
Participants	13*	(a) Report numbers of individuals at each stage of study—eg numbers potentially eligible, examined for eligibility, confirmed eligible, included in the study, completing follow-up, and analysed<br>(b) Give reasons for non-participation at each stage<br>(c) Consider use of a flow diagram	7
Descriptive data	14*	(a) Give characteristics of study participants (eg demographic, clinical, social) and information on exposures and potential confounders<br>(b) Indicate number of participants with missing data for each variable of interest<br>(c) Summarise follow-up time (eg, average and total amount)	7
Outcome data	15*	Report numbers of outcome events or summary measures over time	7

For peer review only

Main results	16	(*a*) Give unadjusted estimates and, if applicable, confounder-adjusted estimates and their precision (eg, 95% confidence interval). Make clear which confounders were adjusted for and why they were included<br>(*b*) Report category boundaries when continuous variables were categorized<br>(*c*) If relevant, consider translating estimates of relative risk into absolute risk for a meaningful time period	-
Other analyses	17	Report other analyses done—eg analyses of subgroups and interactions, and sensitivity analyses	-
**Discussion**			
Key results	18	Summarise key results with reference to study objectives	9
Limitations	19	Discuss limitations of the study, taking into account sources of potential bias or imprecision. Discuss both direction and magnitude of any potential bias	9-12
Interpretation	20	Give a cautious overall interpretation of results considering objectives, limitations, multiplicity of analyses, results from similar studies, and other relevant evidence	13
Generalisability	21	Discuss the generalisability (external validity) of the study results	13
**Other information**			
Funding	22	Give the source of funding and the role of the funders for the present study and, if applicable, for the original study on which the present article is based	14

*Give information separately for exposed and unexposed groups.

Note: An Explanation and Elaboration article discusses each checklist item and gives methodological background and published examples of transparent reporting. The STROBE checklist is best used in conjunction with this article (freely available on the Web sites of PLoS Medicine at http://www.plosmedicine.org/, Annals of Internal Medicine at http://www.annals.org/, and Epidemiology at http://www.epidem.com/). Information on the STROBE Initiative is available at http://www.strobe-statement.org.

For peer review only
